# DDRGK1-mediated ER-phagy attenuates acute kidney injury through ER-stress and apoptosis

**DOI:** 10.1038/s41419-024-06449-4

**Published:** 2024-01-17

**Authors:** Haijiao Jin, Yuanting Yang, Xuying Zhu, Yin Zhou, Yao Xu, Jialin Li, Chaojun Qi, Xinghua Shao, Jingkui Wu, Shan Wu, Hong Cai, Leyi Gu, Shan Mou, Zhaohui Ni, Shu Li, Qisheng Lin

**Affiliations:** 1 Department of Nephrology, Molecular Cell Lab for Kidney Disease, Shanghai Peritoneal Dialysis Research Center, Ren Ji Hospital, Uremia Diagnosis and Treatment Center, Shanghai Jiao Tong University School of Medicine, Shanghai, 200127 China; 2 Department of Nephrology, Shuguang Hospital Affiliated to Shanghai University of Traditional Chinese Medicine, Shanghai, 201200 China; 3 Department of Endoscopy, Shanghai Sixth People’s Hospital Affiliated to Shanghai Jiao Tong University School of Medicine, Shanghai, 200233 China

**Keywords:** Acute kidney injury, Autophagy

## Abstract

Acute kidney injury (AKI) constitutes a prevalent clinical syndrome characterized by elevated morbidity and mortality rates, emerging as a significant public health issue. This study investigates the interplay between endoplasmic reticulum (ER) stress, unfolded protein response (UPR), and ER-associated degradation (ER-phagy) in the pathogenesis of AKI. We employed four distinct murine models of AKI—induced by contrast media, ischemia–reperfusion injury, cisplatin, and folic acid—to elucidate the relationship between ER-phagy, ER stress, and apoptosis. Our findings reveal a marked decrease in ER-phagy coinciding with an accumulation of damaged ER, elevated ER stress, and increased apoptosis across all AKI models. Importantly, overexpression of DDRGK1 in HK-2 cells enhanced ER-phagy levels, ameliorating contrast-induced ER stress and apoptosis. These findings unveil a novel protective mechanism in AKI, wherein DDRGK1–UFL1-mediated ER-phagy mitigates ER stress and apoptosis in renal tubular epithelial cells. Our results thereby contribute to understanding the molecular underpinnings of AKI and offer potential therapeutic targets for its treatment.

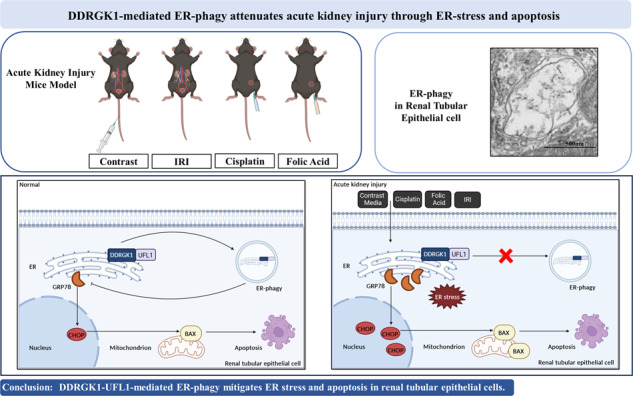

## Introduction

Acute kidney injury (AKI) is a pervasive clinical syndrome affecting 19.3–31% of hospitalized patients globally, posing significant public health challenges [1, 2]. Despite the identification of multiple pathogenic mechanisms, such as ischemia–reperfusion injury (IRI), drug toxicity, and sepsis, the etiological basis of AKI remains largely elusive [3]. Recent advances have highlighted the role of mitochondrial dysfunction, autophagy, and reactive oxygen species (ROS) in AKI [4]; however, the molecular mechanisms underlying its pathogenesis warrant further elucidation for effective clinical intervention.

Emerging research has started to focus on the role of endoplasmic reticulum (ER) in kidney diseases [5]. ER is central to protein synthesis, transport, and posttranslational modification, in addition to serving as a reservoir for intracellular calcium [6]. ER stress, triggered by the accumulation of unfolded and misfolded protein riggers glucose-related protein 78 (GRP78, also known as binding immunoglobulin protein, BiP), activates the unfolded protein response (UPR) through sensors like protein kinase R‐like ER kinase (PERK), inositol requiring enzyme 1‐α (IRE1‐α), and activating transcription factor 6 (ATF6) [7, 8]. This leads to the upregulation of CCAAT/enhancer-binding protein homologous protein (CHOP), a major facilitator of programmed cell death, thereby contributing to kidney disease progression, including AKI [4, 9].

Autophagy selectively degrades damaged or dysfunctional organelles to maintain cellular homeostasis, which is mostly considered to protect against AKI [10, 11]. Selective autophagy, or ‘ER-phagy,’ is a crucial compensatory mechanism for maintaining ER homeostasis [12]. While our previous work has elucidated the protective role of PINK1–PARK2- and BNIP3-mediated mitophagy in cisplatin and contrast-induced AKI [13–15], the role of ER-phagy remains unknown in kidney research. Prior research has established that overexpression of FAM134B leads to ER fragmentation and subsequent lysosomal degradation, as cited in reference [16]. Similarly, TEX264’s interaction with LC3 and GABARAP family proteins, as noted in reference [17], highlights its role in initiating ER-phagy, positioning both FAM134B and TEX264 as key biomarkers of ER-phagy. Moreover, recent advancements using ER-phagy reporter systems and genome-wide CRISPRi screening have revealed that DDRGK1 facilitates the migration of UFL1 to the ER surface, triggering the UFMylation process with ubiquitin fold modifier 1 (UFM1), thereby initiating ER-phagy and the degradation of damaged ER. This process is akin to the PINK1–PARK2 pathway in mitophagy [18]. In kidney study, Deng et al. demonstrated DDRGK1 and unfolded protein response contributes to ER-phagy in ochratoxin A-induced nephrotoxicity [19].

In the present study, we investigate the functional implications of ER-phagy in four distinct mouse models of AKI: ischemia–reperfusion, cisplatin, contrast, and folic acid-induced. Additionally, we explore the relationship between ER-phagy upregulation and apoptosis through DDRGK1 overexpression in vitro. This study aims to unveil a novel mechanism underlying AKI, offering fresh avenues for therapeutic interventions.

## Result

### Evidence of ER-phagy in renal tubular epithelial cells

**Fig. 1:**
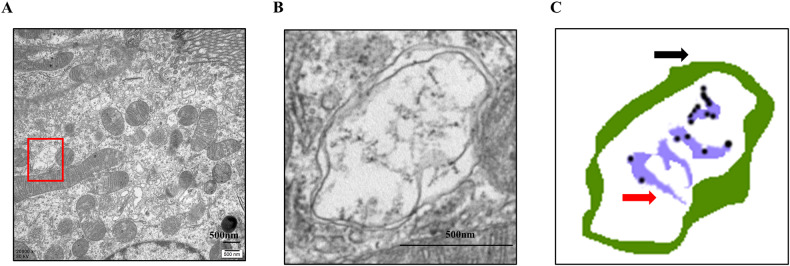
Evidence of ER-phagy in renal tubular epithelial cells. **A** TEM image of renal tubular epithelial cell. **B** The autophagosome involved in ER-phagy. **C** The localization pattern of autophagosome in ER-phagy (the black arrow represents autophagosome membrane, the red arrow represents ER lumen, and the black point represents ribosomes on the surface of rough ER). Scale bar: 500 nm.

To ascertain the presence of ER-phagy in renal resident cells, we preserved electron microscopy (EM) specimens of kidney tissue [6]. As illustrated in Fig. 1, the ER lumen (indicated by red arrows) was found within a bilayer membrane structure (marked by black arrows), confirming the occurrence of ER-phagy in renal tubular epithelial cells.

### Contrast media suppresses ER-phagy, exacerbating ER stress and apoptosis

**Fig. 2:**
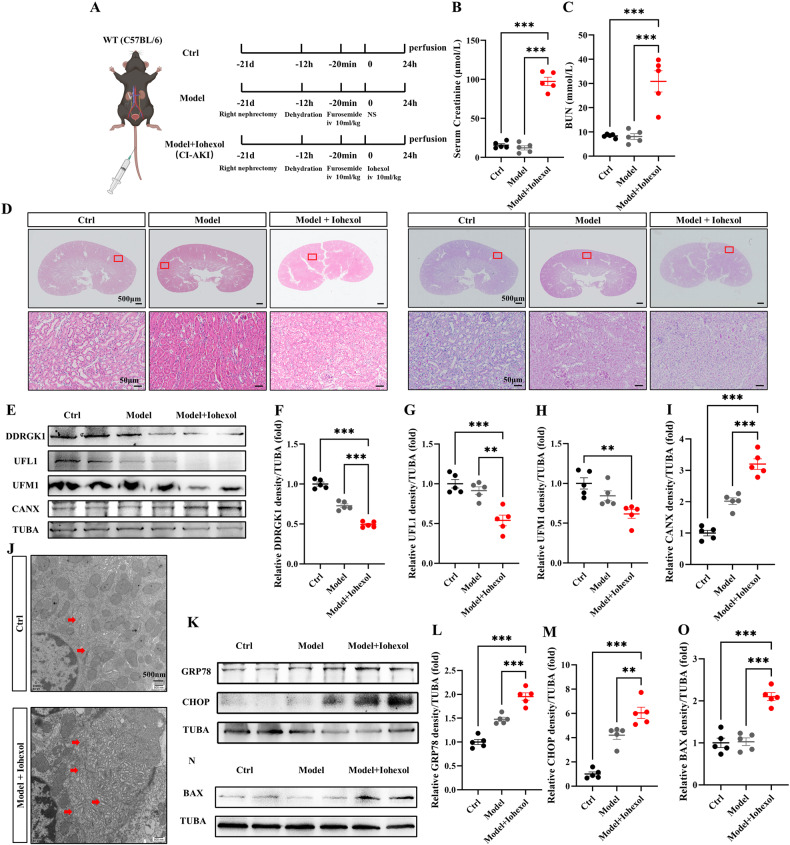
Contrast media suppresses ER-phagy, exacerbating ER stress and apoptosis. **A** Diagrammatic representation of CI-AKI mice (Model + Iohexol, 10 ml/kg), negative control mice (Model + NS), and control mice. The renal function was evaluated by serum creatinine (**B**) and BUN (**C**). **D** Representative histology of HE and PAS staining in the renal cortex. Scale bar: 500 μm and 50 μm. **E**–**I** The immunoblot analysis and quantification of DDRGK1, UFL1, UFM1, CANX in kidney lysates. **J** TEM images of ER in renal tubular epithelial cell. Red arrows: ER. **K**–**M** The immunoblot analysis and quantification of GRP78 and CHOP in kidney lysates. **N**, **O** The immunoblot analysis and quantification of BAX in kidney lysates. Data are presented as the mean ± SEM (*n* = 5). **p < 0.01 and ***p < 0.001.

To delineate the effects of contrast media on ER-phagy in acute kidney injury (AKI), we employed four murine models. Initially, contrast-induced acute kidney injury (CI-AKI) mice were generated as outlined in our preceding investigations (Fig. 2A) [13, 14]. Iohexol (10 ml/kg) was administered via tail vein injection to CI-AKI mice (Model+Iohexol), while an equivalent volume of normal saline (NS) served as a negative control (Model). Both serum creatinine and blood urea nitrogen (BUN) levels elevated significantly in CI-AKI mice compared to Model and Ctrl groups, thereby validating the CI-AKI model (Fig. 2B, C). The histopathological analysis further corroborated the pronounced damage characteristic of CI-AKI, including intraepithelial vacuolar degeneration and interstitial inflammation, in CI-AKI mice relative to controls (Fig. 2D). Immunoblot assays revealed a decline in DDRGK1, UFL1, UFM1, FAM134B, and TEX264 levels accompanied by an increase in calnexin (CANX), an ER marker, in CI-AKI kidney lysates, indicating a decrease in DDRGK1–UFL1-mediated ER-phagy in this model (Fig. 2E–I, Fig. S1). Transmission electron microscopy (TEM) depicted increased ER accumulation in the renal tubular epithelial cells of CI-AKI mice compared to controls, suggesting impaired ER clearance due to reduced ER-phagy (Fig. 2J). Additionally, elevated levels of GRP78 and CHOP in CI-AKI kidneys indicated exacerbated ER stress (Fig. 2K–M). A corresponding increase in the pro-apoptotic BAX protein was also observed in CI-AKI kidneys (Fig. 2N, O).

### IRI inhibits ER-phagy, intensifying ER stress and apoptosis

**Fig. 3:**
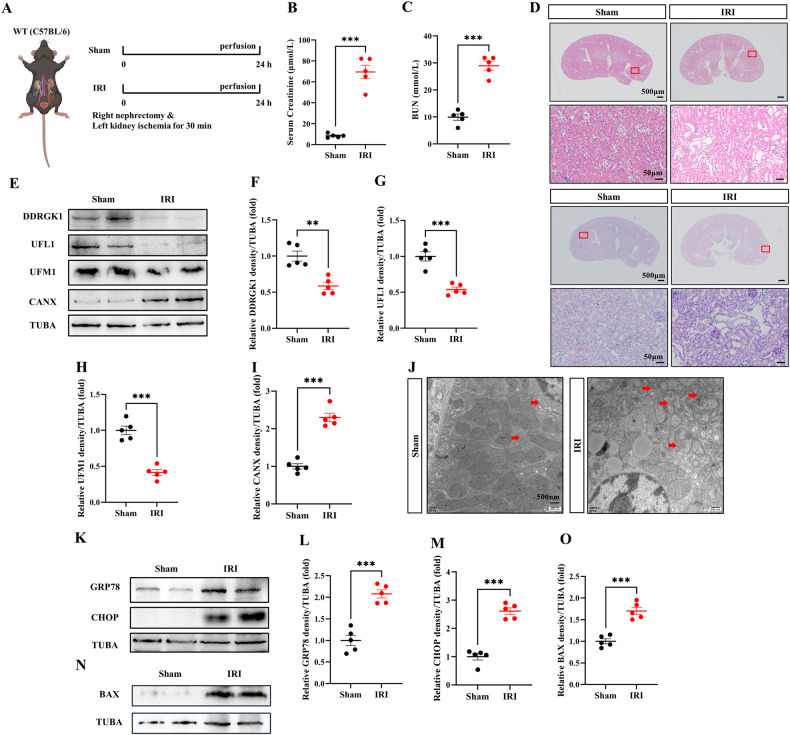
IRI inhibits ER-phagy, intensifying ER stress and apoptosis. **A** Diagrammatic representation of IRI mice (right nephrectomy and left kidney ischemia 30 min) and sham mice. The renal function was evaluated by serum creatinine (**B**) and BUN (**C**). **D** Representative histology of HE and PAS staining in the renal cortex. Scale bar: 500 μm and 50 μm. **E**–**I** The immunoblot analysis and quantification of DDRGK1, UFL1, UFM1, CANX in kidney lysates. **J** TEM images of ER in renal tubular epithelial cell. Red arrows: ER. **K**–**M** The immunoblot analysis and quantification of GRP78 and CHOP in kidney lysates. **N**, **O** The immunoblot analysis and quantification of BAX in kidney lysates. Data are presented as the mean ± SEM (*n* = 5). ***p < 0.001.

Subsequently, a renal IRI model was generated as previously described [20]. Briefly, mice underwent right nephrectomy followed by 30-min ischemia of the left kidney post-anesthetization; tissues and blood were collected 24 h post-procedure (Fig. 3A). Elevated serum creatinine and BUN, along with exacerbated histological damage such as extensive necrosis of tubular epithelial cells and loss of brush border, were observed in IRI mice (Fig. 3B–D). In the kidney lysates of these IRI mice, levels of DDRGK1, UFL1, UFM1, FAM134B, and TEX264 were decreased while CANX was increased, indicating inhibition of ER-phagy post-IRI (Fig. 3E–I, Fig. S2). Consequently, an accumulation of damaged ER was observed in renal tubular epithelial cells following IRI (Fig. 3J). Immunoblot analyses of GRP78, CHOP, and BAX demonstrated heightened ER stress and apoptosis post-IRI (Fig. 3K–O).

### Cisplatin negatively regulates ER-phagy, heightening ER stress and apoptosis

**Fig. 4:**
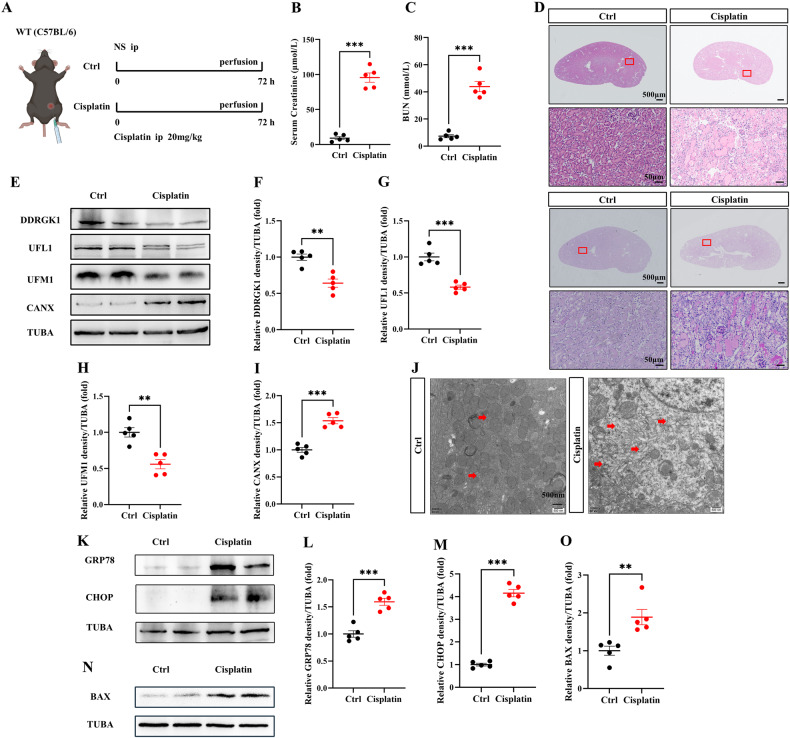
Cisplatin negatively regulates ER-phagy, heightening ER stress and apoptosis. **A** Diagrammatic representation of Cisplatin mice (20 mg/kg) and control mice. The renal function was evaluated by serum creatinine (**B**) and BUN (**C**). **D** Representative histology of HE and PAS staining in the renal cortex. Scale bar: 500 μm and 50 μm. **E**–**I** The immunoblot analysis and quantification of DDRGK1, UFL1, UFM1, and CANX in kidney lysates. **J** TEM images of ER in renal tubular epithelial cell. Red arrows: ER. **K**–**M** The immunoblot analysis and quantification of GRP78 and CHOP in kidney lysates. **N**, **O** The immunoblot analysis and quantification of BAX in kidney lysates. Data are presented as the mean ± SEM (*n* = 5). **p < 0.01 and ***p < 0.001.

A cisplatin-induced AKI murine model was generated, which is consistent with our earlier studies (Fig. 4A) [15]. Following intraperitoneal injection of cisplatin (20 mg/kg) for 72 h, a significant increase in serum creatinine and BUN was observed (Fig. 4B, C). HE and PAS staining unveiled enhanced intraepithelial vacuolar degeneration and cast formation in cisplatin-treated mice (Fig. 4D). Immunoblot analyses revealed diminished DDRGK1, UFL1, UFM1, FAM134B, and TEX264 levels and elevated CANX, signifying a decrease in ER-phagy in cisplatin-induced AKI (Fig. 4E–I, Fig. S3). TEM indicated ER expansion and accumulation in renal tubular epithelial cells post-cisplatin treatment (Fig. 4J). Elevated levels of ER stress markers GRP78 and CHOP were detected following cisplatin-induced ER-phagy inhibition (Fig. 4K–M). An increase in apoptosis, as indicated by elevated BAX protein levels, was also noted in cisplatin-induced AKI (Fig. 4N, O).

### Folic acid suppresses ER-phagy, worsening ER stress and apoptosis

**Fig. 5:**
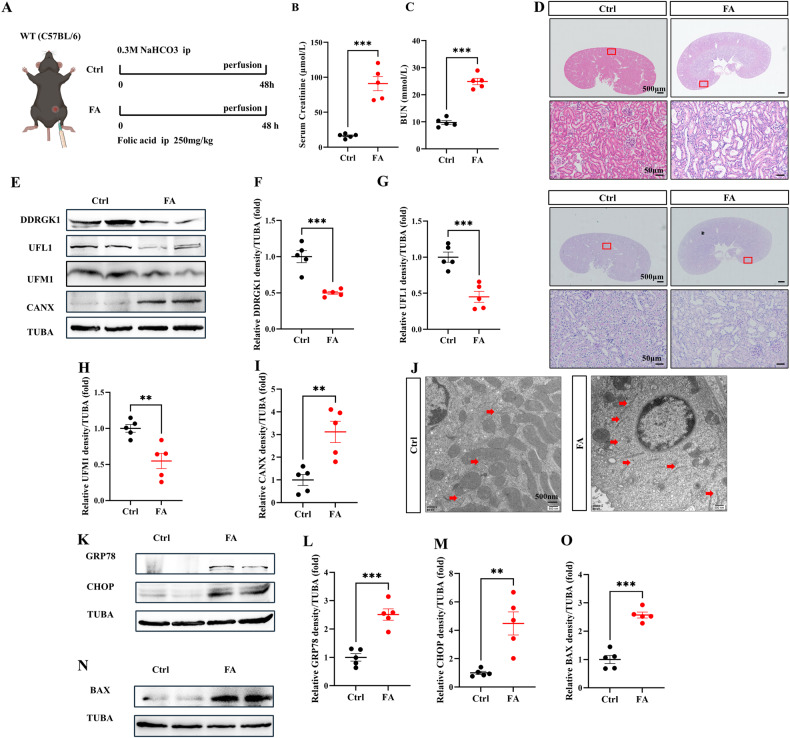
Folic acid suppresses ER-phagy, worsening ER stress, and apoptosis. **A** Diagrammatic representation of FA mice (250 mg/kg) and control mice. The renal function was evaluated by serum creatinine (**B**) and BUN (**C**). **D** Representative histology of HE and PAS staining in the renal cortex. Scale bar: 500 μm and 50 μm. **E**–I The immunoblot analysis and quantification of DDRGK1, UFL1, UFM1, and CANX in kidney lysates. **J** TEM images of ER in renal tubular epithelial cell. Red arrows: ER. **K**–**M** The immunoblot analysis and quantification of GRP78 and CHOP in kidney lysates. **N**, **O** The immunoblot analysis and quantification of BAX in kidney lysates. Data are presented as the mean ± SEM (*n* = 5). **p < 0.01 and ***p < 0.001.

Folic acid (250 mg/kg) was dissolved in 0.3 M sodium bicarbonate and administered to mice (Fig. 5A) [21]. Renal function in folic acid-induced AKI was assessed by serum creatinine and BUN levels (Fig. 5B, C). Concurrent with histological injuries, such as loss of brush border and tubular dilation (Fig. 5D), we confirmed folic acid-induced renal injury in this mouse model. Immunoblot assays of DDRGK1, UFL1, UFM1, FAM134B, TEX264, and CANX revealed folic acid-mediated downregulation of ER-phagy in kidney lysates (Fig. 5E–I, Fig. S4). TEM analyses indicated ER expansion and impaired clearance (Fig. 5J). Elevated levels of GRP78, CHOP, and BAX substantiated folic acid-induced ER stress and apoptosis in renal tubular epithelial cells (Fig. 5K–O).

### In vitro CI-AKI model shows reduced ER-phagy

**Fig. 6:**
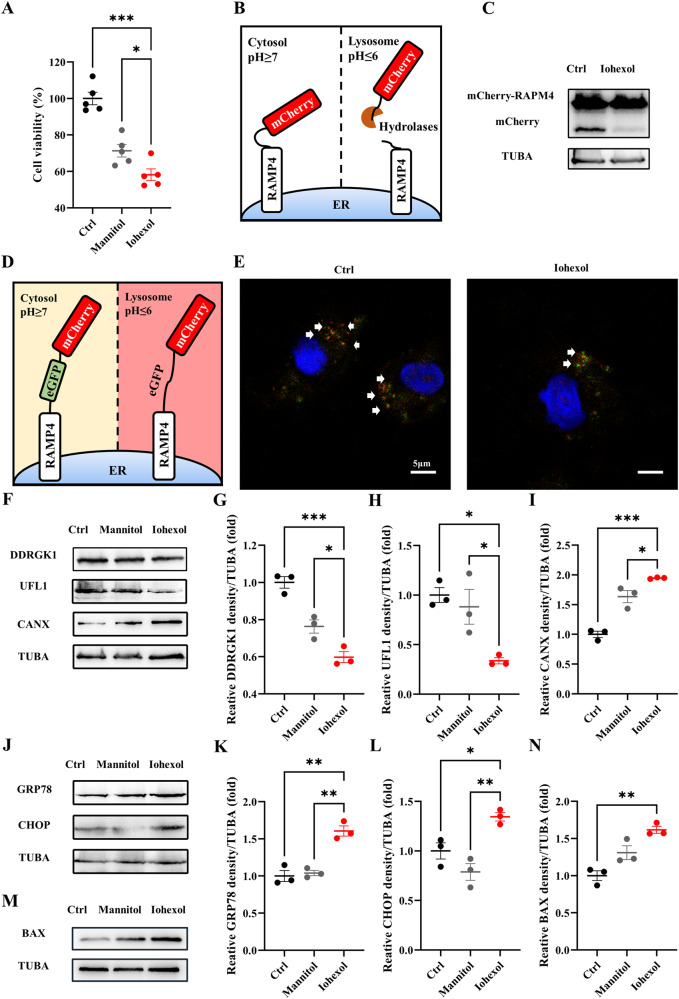
In vitro CI-AKI model shows reduced ER-phagy. HK-2 cells were incubated in DMEM/F12 containing Iohexol (150 mg I/ml) for 6 h. **A** CCK-8 assay of Iohexol-treated HK-2 cells, together with the isosmotic control group, mannitol-treated HK-2 cells. **B**, **C** Diagrammatic representation of the mCherry cleavage from ER assay. Briefly, degradation of ER resulted in the lysosomal cleavage of the mCherry tag from RAMP4, which caused a smaller, mCherry-only product to be resolved by immunoblot analysis. **D** Diagrammatic representation of ER-phagy tandem reporter assay. eGFP is quenched in lysosomal-induced low pH situations, causing GFP^+^/mCherry^+^ to GFP^−^/mCherry^+^ during ER-phagy. **E** The representative image of ER-phagy tandem reporter assay by confocal microscope. Scale bar: 5 μm. White arrows: GFP^−^/mCherry^+^ puncta. **F**–**I** The immunoblot analysis and quantification of DDRGK1, UFL1, CANX in cell lysates. **J**–**L** The immunoblot analysis and quantification of GRP78 and CHOP in cell lysates. **M**, **N** The immunoblot analysis and quantification of BAX in cell lysates. Data are presented as the mean ± SEM (*n* = 3). *p < 0.05, **p < 0.01 and ***p < 0.001.

In vitro, HK-2 cells were exposed to Iohexol (150 mgI/mL, 150 mg iodine per mL) for 6 h to generate a contrast media-induced cell model [22]. Cell viability was gauged using a CCK-8 assay (Fig. 6A). To quantify ER-phagy levels, the pLenti-X1-hygro-mCherry-RAMP4 plasmid was introduced into HK-2 cells (Fig. 6B) [23]. Lysosomal degradation of ER led to cleavage of the mCherry tag from RAMP4, yielding a smaller, mCherry-only product detectable by immunoblotting. During nutrient deprivation with Earl’s Balanced Salt Solution (EBSS), the mCherry-only product increased in HK-2 cells but was significantly reduced when treated with Iohexol (Fig. 6C). An alternative plasmid, TetOn-mCherry-eGFP-RAMP4, was also employed for ER-phagy detection (Fig. 6D). Lysosomal acidification led to GFP+/mCherry+ transitioning to GFP–/mCherry+ during ER-phagy [23]. A greater prevalence of GFP–/mCherry+ HK-2 cells was observed in the control group compared to the Iohexol group (Fig. 6E). Immunoblot analyses of DDRGK1, UFL1, and CANX corroborated the downregulation of ER-phagy at the protein level (Fig. 6F–I). Levels of ER stress and apoptosis markers GRP78, CHOP, and BAX further verified reduced ER-phagy and increased stress and apoptosis in contrast to media-treated HK-2 cells (Fig. 6J–N).

### *DDRGK1* overexpression rescues ER-phagy and mitigates ER stress and apoptosis

**Fig. 7:**
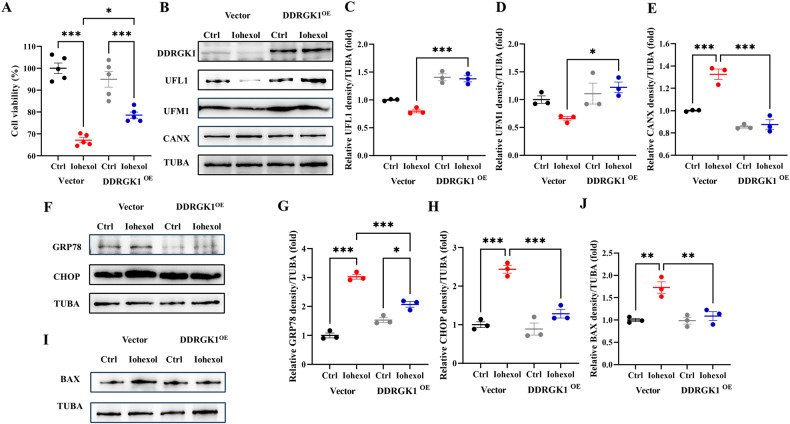
*DDRGK1* overexpression rescues ER-phagy and mitigates ER stress and apoptosis. *DDRGK1* plasmid was transfected to HK-2 cells to overexpress DDRGK1. **A** CCK-8 assay of *DDRGK1* overexpression HK-2 cells with or without Iohexol. **B**–**E** The immunoblot analysis and quantification of DDRGK1, UFL1, UFM1, CANX in cell lysates. **F**–**H** The immunoblot analysis and quantification of GRP78 and CHOP in cell lysates. **I**, **J** The immunoblot analysis and quantification of BAX in cell lysates. Data are presented as the mean ± SEM (*n* = 3). *p < 0.05, **p < 0.01 and ***p < 0.001.

To further delineate the role of DDRGK1–UFL1-mediated ER-phagy in CI-AKI, a DDRGK1 overexpression plasmid was introduced into HK-2 cells. Cell viability was assessed via CCK-8 assay and showed that DDRGK1 overexpression ameliorated HK-2 cell viability post-Iohexol treatment (Fig. 7A). Immunoblot assays confirmed the efficiency of DDRGK1 overexpression (Fig. 7B). Enhanced UFL1, UFM1 and TEX264 levels, and reduced CANX levels indicated upregulated DDRGK1–UFL1-mediated ER-phagy (Fig. 7B–E, Fig. S5). Reduced levels of ER stress markers GRP78 and CHOP substantiated the protective role of DDRGK1–UFL1-mediated ER-phagy against ER stress (Fig. 7F–H). Iohexol-induced elevation of BAX levels was mitigated by DDRGK1 overexpression (Fig. 7I, J), reinforcing the protective role of DDRGK1–UFL1-mediated ER-phagy against apoptosis in HK-2 cells exposed to contrast media.

### *UFL1* overexpression rescues ER-phagy and mitigates ER stress and apoptosis

**Fig. 8:**
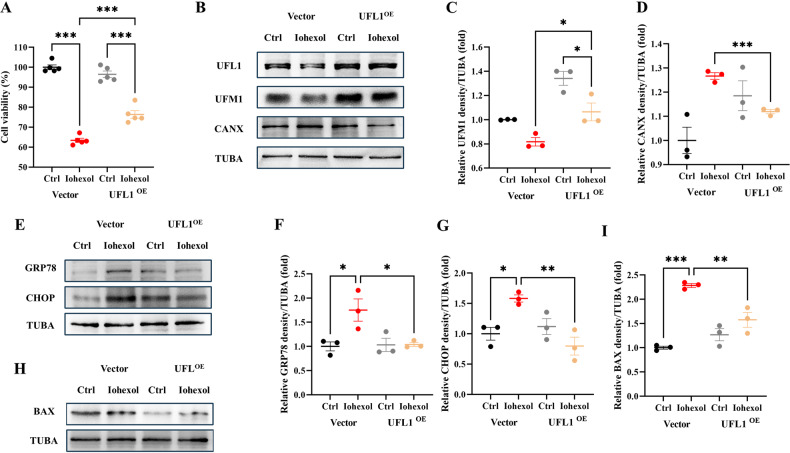
*UFL1* overexpression rescues ER-phagy and mitigates ER stress and apoptosis. *UFL1* plasmid was transfected to HK-2 cells to overexpress UFL1. **A** CCK-8 assay of *UFL1* overexpression HK-2 cells with or without Iohexol. **B**–**D** The immunoblot analysis and quantification of UFL1, UFM1, and CANX in cell lysates. **E**–**G** The immunoblot analysis and quantification of GRP78 and CHOP in cell lysates. **H**, **I** The immunoblot analysis and quantification of BAX in cell lysates. Data are presented as the mean ± SEM (*n* = 3). *p < 0.05, **p < 0.01 and ***p < 0.001.

To further elucidate UFL1-mediated ER-phagy’s role in CI-AKI, we introduced a UFL1 overexpression plasmid into HK-2 cells. Cell viability, assessed via the CCK-8 assay, demonstrated that UFL1 overexpression improved the viability of HK-2 cells post-Iohexol treatment (Fig. 8A). Immunoblot assays confirmed the successful overexpression of UFL1 (Fig. 8B). The upregulation of UFM1 and TEX264 levels and decrease in CANX levels suggest enhanced UFL1-mediated ER-phagy (Fig. 8B–D, Fig. S6). Additionally, the reduced expression of ER stress markers GRP78 and CHOP further supports the protective role of UFL1-mediated ER-phagy against ER stress (Fig. 8E–G). The mitigation of Iohexol-induced elevation in BAX levels by UFL1 overexpression underscores its protective role against apoptosis in HK-2 cells exposed to contrast media (Fig. 8H, I).

## Discussion

**Fig. 9:**
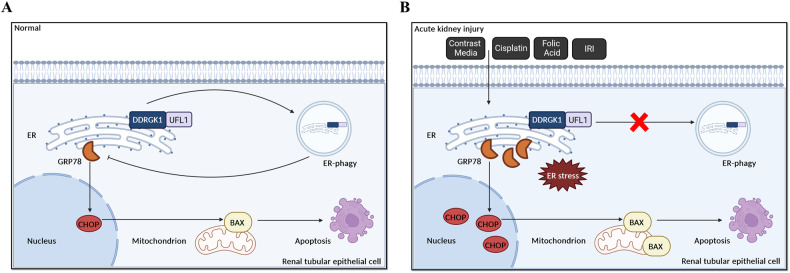
Schematic representation of ER-phagy, ER stress, and apoptosis in acute kidney injury. Under physiological conditions, DDRGK1–UFL1-mediated ER-phagy eliminated ER stress and reduced apoptosis of renal tubular epithelial cells (**A**). During exposure to contrast media, cisplatin, folic acid, or ischemia–reperfusion injury, DDRGK1–UFL1-mediated ER-phagy was reduced, which resulted in aggravation of ER stress and apoptosis of renal tubular epithelial cells (**B**). Thus, ER-phagy protects against acute kidney injury by decreasing ER stress and apoptosis.

In the present study, the impact of ER-phagy was scrutinized in AKI models induced by contrast, IRI, cisplatin, and folic acid. Our findings revealed a decrease in ER-phagy in both AKI mouse models and contrast-induced HK-2 cells, leading to an exacerbation of ER stress and apoptosis in renal tubular epithelial cells. Notably, DDRGK1 or UFL1 overexpression led to the upregulation of UFM1, thereby enhancing ER-phagy and ameliorating ER stress and apoptosis in HK-2 cells (Fig. 9). This study unveils a novel mechanism wherein ER-phagy provides protection against AKI by mitigating ER stress and apoptosis, suggesting that ER-phagy and ER stress may serve as viable therapeutic targets for AKI treatment.

ER stress culminates in the accumulation of UPR elements and subsequently induces apoptosis, a process implicated in the pathogenesis of kidney disease. In the context of unilateral renal IRI, Shu et al. demonstrated that IRI elevated CHOP and GRP78 levels, thereby inducing ER stress and further ameliorating apoptosis and inflammation in post-ischemic kidneys [24]. In lipopolysaccharide (LPS)-induced AKI models, angiopoietin-like protein 3 knockout mice showed alleviated ER stress and apoptosis via the inhibition of the ROS/GRP78/BAX pathway [25]. Forsythiaside A suppressed ER stress-induced inflammation and apoptosis, thereby suggesting a pivotal role of ER stress in sepsis-induced AKI [26, 27]. GRP170, an ER protein regulating the degradation and assembly of the epithelial sodium channel, inhibits UPR and ER stress, which protects against ischemia- and sepsis-induced AKI [28]. CHOP and GRP78 levels were also elevated in conjunction with increased apoptosis in cisplatin-induced AKI models [29, 30]. For chronic kidney disease, Fan et al. confirmed that reticulon 1A modulated ER stress, thereby promoting fibrosis in unilateral ureteral obstruction and elevated proteinuria in diabetic kidney disease models [31]. In this study, we corroborate the involvement of ER stress in IRI and cisplatin-induced AKI and extend these observations to contrast-induced and folic acid-induced AKI models. The results emphasize the role of ER in cell death and the significance of ER stress in AKI studies.

Autophagy, often viewed as a double-edged sword, has traditionally been considered a protective mechanism in AKI. However, persistent activation of autophagy can exacerbate renal interstitial fibrosis [10, 32–34]. Our earlier research concentrated on mitophagy, a form of selective autophagy aimed at eliminating damaged mitochondria, and demonstrated its protective role against contrast and cisplatin-induced AKI through reducing mitochondrial ROS and apoptosis or ferroptosis [13–15]. Sun lab reviewed the role of abnormal ER homeostasis in diabetic kidney disease [35] and supposed ER-phagy might have an effect on kidney diseases. ER is another critical organelle in renal tubular epithelial cells [24], and studies focusing on ER-phagy in kidney diseases are scarce. This investigation is the first to establish that ER-phagy upregulation ameliorates ER stress and apoptosis in AKI, thereby underscoring its protective role. The mechanism of ER-phagy is slightly different from mitophagy in AKI. Mitophagy is irritability elevated in AKI, which indicates the protective role for eliminating mitochondrion after injury [13–15, 36]. Nevertheless, in the present study, we show ER-phagy is reduced in contrast to IRI, cisplatin, and folic acid-induced AKI, and meanwhile, ER stress and apoptosis are aggravated, which suggests that IRI or drugs might directly work on ER and reduce ER-phagy, resulting downstream accumulation of ER stress and renal tubular epithelial cell death in AKI. Our further work will focus on this interesting topic.

ER-phagy, a process dedicated to the selective clearance of ER subdomains, helps maintain ER homeostasis following stress events [12]. Previously recognized ER-phagy receptors, such as Family with sequence similarity 134, member B (FAM134B), testis expressed 264 (TEX264), and reticulon 3 (RTN3), were identified as targeting autophagic membranes on the ER surface through C-terminal or N-terminal LC3­interacting region (LIR) motif [37]. A recent genome-wide CRISPR–Cas9 screen revealed that DDRGK1 recruits UFL1 to the ER surface, triggering UFM1 and UFMylation of ER-resident proteins and initiating ER-phagy, akin to PINK1–Parkin-mediated mitophagy [18]. In this study, we demonstrate that DDRGK1–UFL1-mediated ER-phagy is diminished in AKI models induced by contrast, IRI, cisplatin, and folic acid. Additionally, DDRGK1 overexpression in HK-2 cells ameliorated apoptosis and improved cell viability in vitro. Our results confirm the DDRGK1–UFL1-mediated ER-phagy protects against IRI- and drug-induced AKI.

In previous mechanistic studies, DDRGK1 is shown to recruit UFL1 to the ER surface, and their synergistic ER-resident activity with UFM1 leads to a reduction of monomeric UFM1 in HCT116 CRISPRi cells [18]. Contrastingly, mammalian studies present different outcomes. For instance, in mouse models of transverse aortic constriction-induced cardiac hypertrophy, both UFL1 and UFM1 are upregulated, activating protein UFMylation [38]. In ketosis-induced cow liver injury, there’s a reduction in DDRGK1-dependent UFMylation, accompanied by a decrease in UFM1 [39]. Similarly, in nonalcoholic fatty liver disease in mice, there’s an upregulation of DDRGK1, UFM1, and UFM1-conjugated DDRGK1, contributing to increased UFMylation expression [40]. Our study further corroborates these findings, showing a reduction in DDRGK1, UFL1, and UFM1 in acute kidney injury, which suggests a decrease in ER-phagy. The varying levels of UFM1 in DDRGK1–UFL1-mediated UFMylation across different studies are noteworthy. Previous research indicates a reduction in UFM1 levels in the crypts of both Ddrgk1knockout and Ufl1 knockout mice, suggesting that DDRGK1 and UFL1 not only contribute to UFM1’s conjugation to the ER but also possibly influence UFM1 levels [41]. Future research is essential to explore this mechanism further in mammals.

In summary, our study reveals a decrease in ER-phagy in various AKI models, accompanied by an upregulation of ER stress and apoptosis in renal tubular epithelial cells. Furthermore, DDRGK1–UFL1-mediated ER-phagy overexpression alleviates contrast-induced ER stress and apoptosis in HK-2 cells. These findings suggest that ER-phagy could be a promising therapeutic target for the clinical prevention and treatment of AKI.

## Methods

### Animal models and induction of acute kidney injury (AKI)

C57BL/6J male mice (6–8 weeks, 20–25 g) were purchased from SPF (Beijing) Biotechnology Co., Ltd. Experimental protocols were endorsed by the Animal Care Committee of Ren Ji Hospital and Shanghai Jiao Tong University School of Medicine.

For CI-AKI mice, the induction process was performed as delineated in our prior studies [13, 14]. Briefly, mice who underwent right nephrectomy post-anesthesia were subjected to normal feeding for 3 weeks, followed by 12-h dehydration. Subsequently, they received an intravenous injection of furosemide (Sigma-Aldrich, F4381; diluted to 10 mg/mL, 10 mL/kg body weight) and then Iohexol (GE Healthcare, Omnipaque 350; 10 mL/kg body weight) to form the Model+Iohexol group. Model mice were administered normal saline (NS) as negative controls. All animals were euthanized after 24 h for serum and kidney tissue collection.

IRI mice were induced as previously described [20]. Post-anesthesia, the mice underwent right nephrectomy, and arterial clamps were applied to the left kidney vessels for 30 min. Sham mice received sham surgeries. All animals were sacrificed at 24 h for tissue collection.

Cisplatin mice were treated as outlined in our previous work [15]. Mice were administered cisplatin (Jiangsu Hansoh, H20040813, 20 mg/kg body weight) via intraperitoneal injection. Control mice received equivalent volumes of NS. All subjects were euthanized at 72 hours for serum and kidney tissue retrieval.

FA mice were induced as previously detailed [21]. Mice received intraperitoneal injections of folic acid (MCE, HY-16637, diluted in 0.3 M sodium bicarbonate, 250 mg/kg body weight). Control mice received equivalent volumes of 0.3 M sodium bicarbonate. All subjects were euthanized at 48 h for tissue collection.

### Cell culture and treatments

Human renal proximal tubular cells (HK-2) were acquired from ATCC (ATCC® CRL-2190) and cultivated in DMEM/F-12 medium (ThermoFisher Scientific, 11330057), supplemented with 1% penicillin/streptomycin (ThermoFisher Scientific, 15140122) and 10% fetal bovine serum (ThermoFisher Scientific, 10099158). HK-2 cells were transfected with a DDRGK1 plasmid (1 μg/μL) for 8 h using Lipofectamine™ 3000 (Thermo Fisher Scientific, L3000150) and were subsequently treated with Iohexol (150 mgI/mL) for 6 h post-transfection [22].

The DDRGK1 overexpression plasmid (Human, gene ID: 65992) and UFL1 overexpression plasmid (Human, gene ID: 51569) were engineered by GeneChem Company, cloned into the pGV740 vector, and their sequence was verified through DNA sequencing.

### Renal function and histopathological analysis

Renal function was assessed by measuring serum creatinine and BUN levels using kits from Nanjing Jiancheng Bioengineering Institute (C011-2-1 for creatinine and C013-2-1 for BUN), according to manufacturer guidelines. PAS and HE staining were performed as previously delineated [42].

### Immunoblotting

Proteins were resolved on 10–12% SDS-PAGE gels, as previously described [15], and transferred to PVDF membranes (0.45 μm). Membranes were blocked with 5% skim milk and incubated with primary antibodies (1:1000) at 4 °C overnight, followed by secondary antibodies (1:3000) for 1 h at room temperature. Densitometric analysis was executed using ImageJ software. The density of the target proteins was normalized to that of TUBA.

Primary antibodies were listed as following: DDRGK1 (ProteinTech, 21445-1-AP), UFL1 (novus biologicals, NBP1-79039), UFM1(abcam, ab109305), CANX (Cell Signaling, 2679), GRP78 (Cell Signaling, 3177), CHOP (Cell Signaling, 2895), BAX (ProteinTech, 50599-2-Ig), mCherry (Abcam, ab183628), FAM134B (Cell Signaling, 83414), TEX264 (ProteinTech, 25858-1-AP) and TUBA (Beyotime, AF0001). Secondary antibodies were listed as follows: HRP-labeled Goat Anti-Rabbit IgG (Beyotime, A0208) and HRP-labeled Goat Anti-Mouse IgG (Beyotime, A0216).

### Transmission electron microscopy (TEM)

The kidney samples were immediately fixed in 2% glutaraldehyde and processed for TEM by the Core Facility of Basic Medical Sciences, Shanghai Jiao Tong University of Medicine, as previously described [15]. Sections were observed under a Hitachi H-7650 transmission electron microscope.

### Cell viability analysis

Cell viability was evaluated using a CCK-8 assay (Dojindo, CK04). After treatment with Iohexol, HK-2 cells were incubated with 10 μL of CCK-8 solution at 37 °C, and absorbance at 450 nm was measured every 30 min using a BioTak CytationTM^3^reader.

### In vitro ER-phagy assay

ER-phagy was assessed through mCherry cleavage and ER-phagy tandem reporter assays, as previously outlined [23]. HK-2 cells were transfected with pLenti-X1-hygro-mCherry-RAMP4 (Addgene, 118391) for mCherry cleavage assays and with TetOn-mCherry-eGFP-RAMP4 (Addgene, 109014) for ER-phagy tandem reporter assays. Observations were made using a ZEISS 710 confocal microscope after cells were starved in EBSS (Invitrogen, 24010043) for 2 h prior to Iohexol exposure.

### Statistical analysis

Statistical analyses were conducted using GraphPad Prism 8. Data are presented as mean ± SEM. One-way ANOVA and Tukey’s post hoc test were utilized to compare multiple groups, and Student’s t-test was used to compare two groups. A *P*-value < 0.05 was considered statistically significant.

### Reporting summary

Further information on research design is available in the Nature Research Reporting Summary linked to this article.

### Supplementary information

Authorship Change Approval

Supplementary File-Original western blots

Supplemental Figure 1-6

Reporting Summary

## Data Availability

The authors declare that all data in the article is available.
