# Supplementary material for: DDRGK1-mediated ER-phagy attenuates acute kidney injury through ER-stress and apoptosis
Source: Cell Death Dis. 2024 Jan 17;15(1):63. doi: 10.1038/s41419-024-06449-4 (PMC10794694; doi:10.1038/s41419-024-06449-4)
Supplement: Supplementary file 1 — Authorship Change Approval [file 41419_2024_6449_MOESM1_ESM.pdf]

# Authorship Change Approval

Haijiao Jin

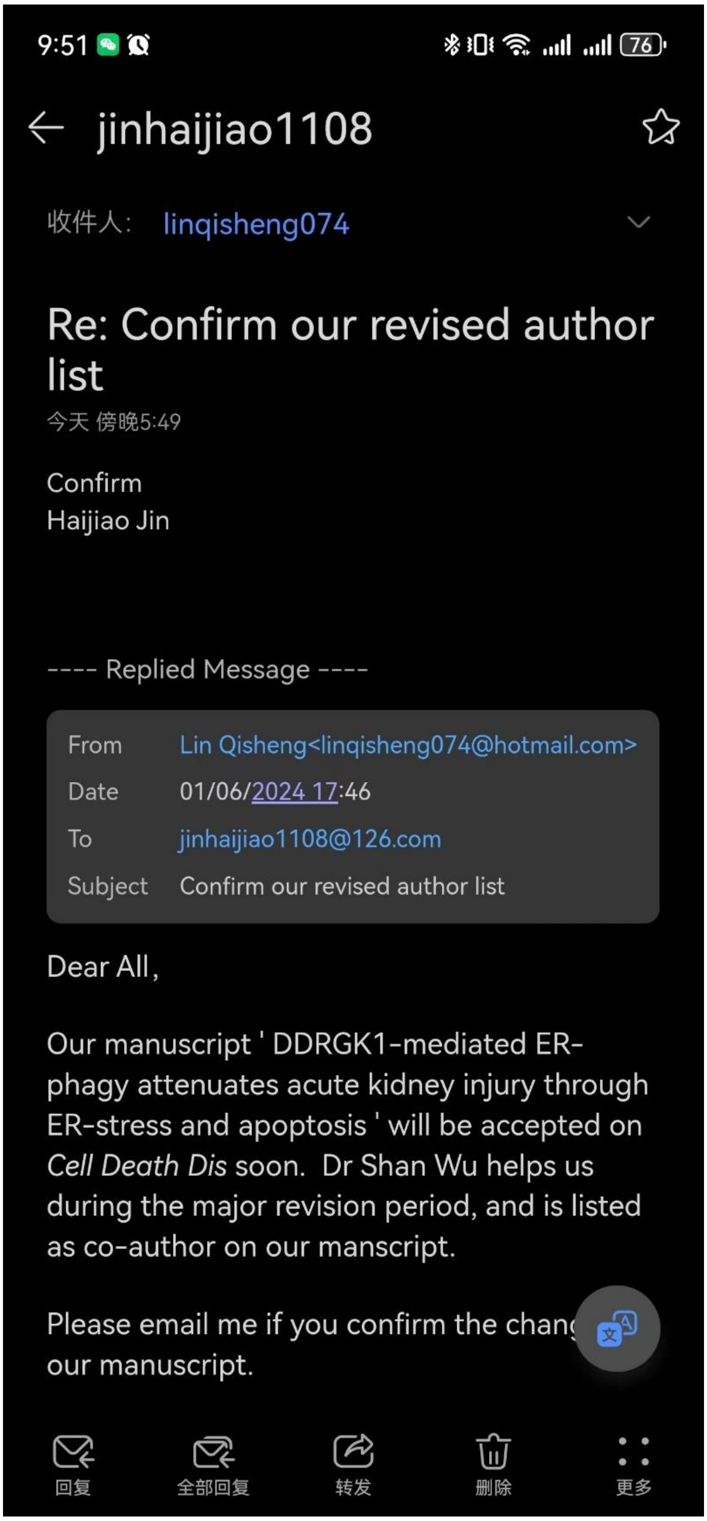

9:51 76

jinhaijiao1108

收件人: linqisheng074

Re: Confirm our revised author list

今天 傍晚5:49

Confirm
Haijiao Jin

---- Replied Message ----

| | |
|---|---|
| From | Lin Qisheng<linqisheng074@hotmail.com> |
| Date | 01/06/2024 17:46 |
| To | jinhaijiao1108@126.com |
| Subject | Confirm our revised author list |

Dear All,

Our manuscript ' DDRGK1-mediated ER-phagy attenuates acute kidney injury through ER-stress and apoptosis ' will be accepted on *Cell Death Dis* soon. Dr Shan Wu helps us during the major revision period, and is listed as co-author on our manscript.

Please email me if you confirm the chang[illegible] our manuscript.

回复 全部回复 转发 删除 更多

Yuanting Yang

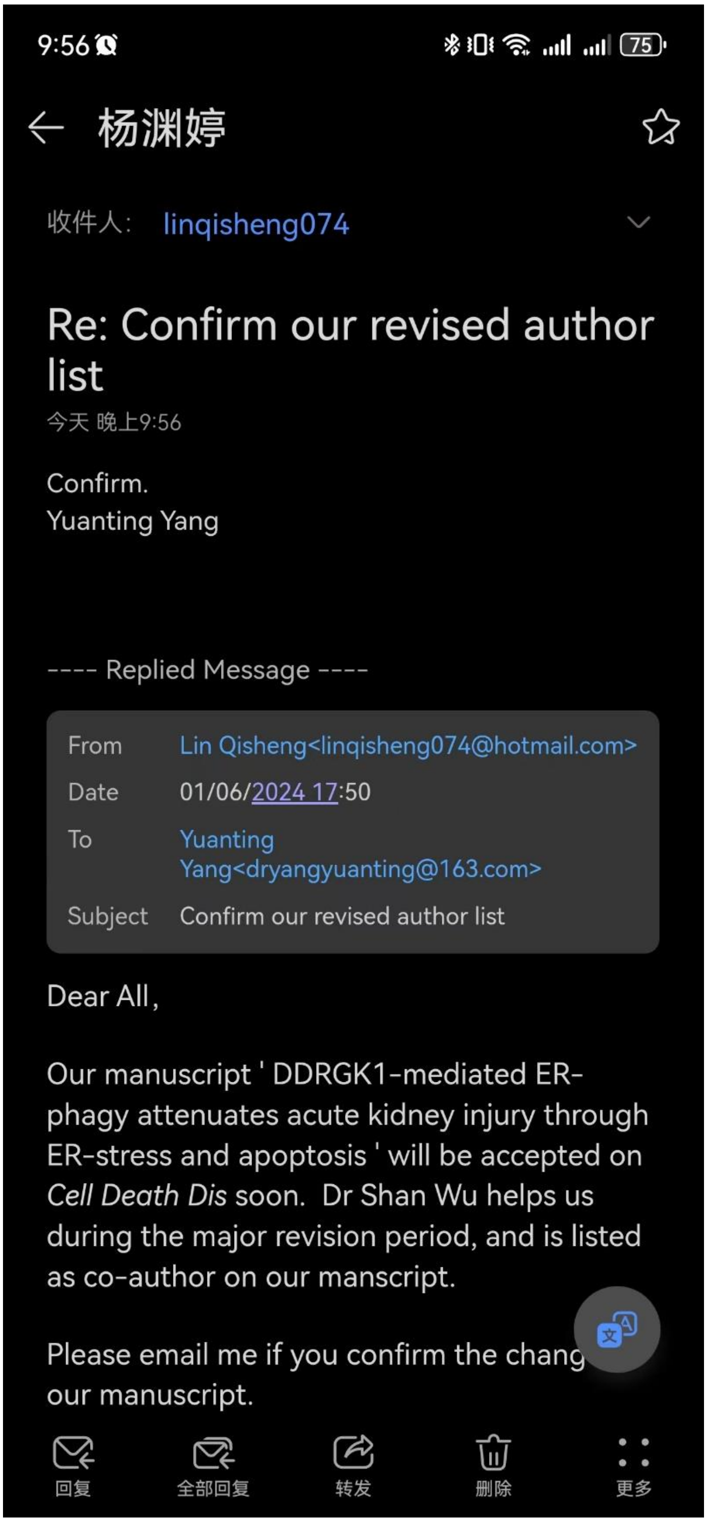

9:56

← 杨渊婷

收件人: linqisheng074

Re: Confirm our revised author list

今天 晚上9:56

Confirm.
Yuanting Yang

---- Replied Message ----

| | |
|---|---|
| From | Lin Qisheng<linqisheng074@hotmail.com> |
| Date | 01/06/2024 17:50 |
| To | Yuanting Yang<dryangyuanting@163.com> |
| Subject | Confirm our revised author list |

Dear All,

Our manuscript ' DDRGK1-mediated ER-phagy attenuates acute kidney injury through ER-stress and apoptosis ' will be accepted on *Cell Death Dis* soon. Dr Shan Wu helps us during the major revision period, and is listed as co-author on our manscript.

Please email me if you confirm the chang[illegible] our manuscript.

回复 全部回复 转发 删除 更多

Xuying Zhu

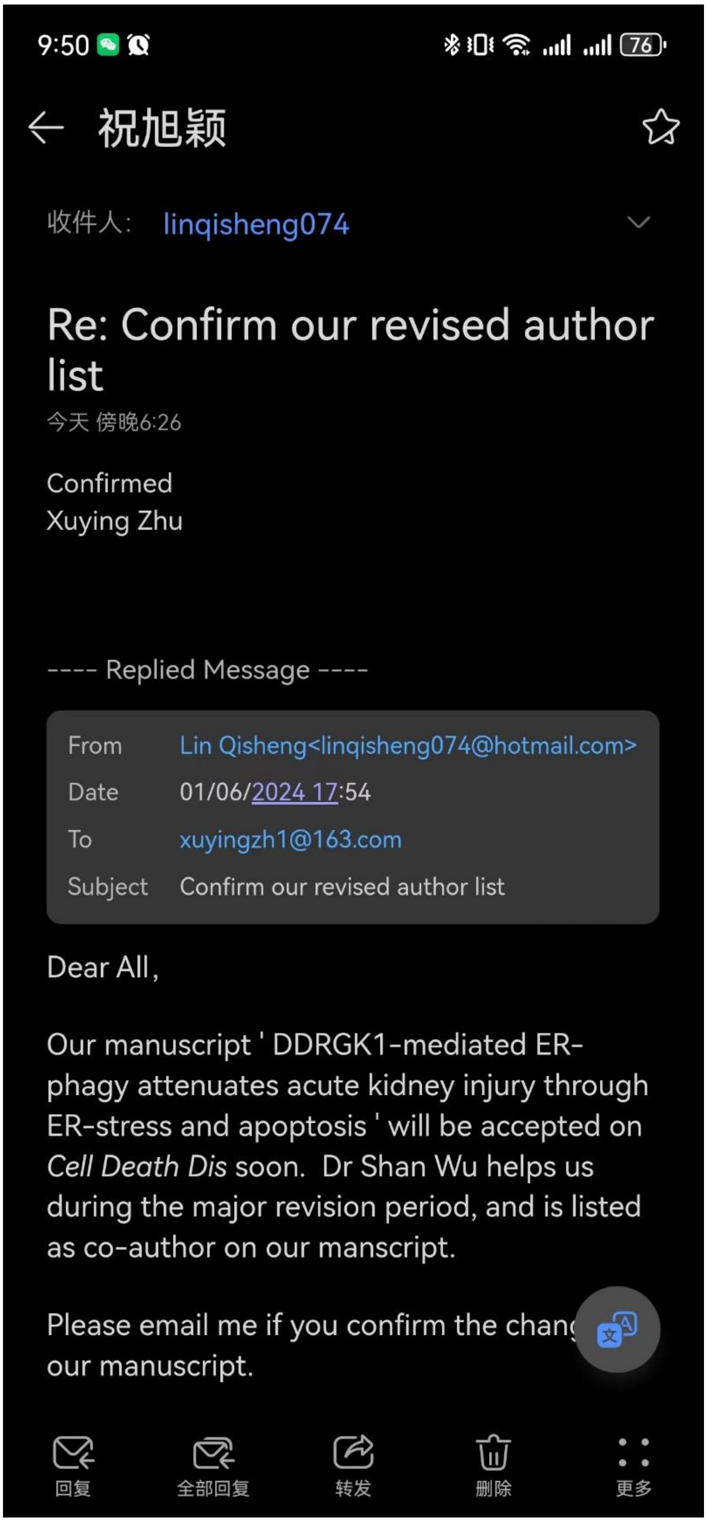

9:50

祝旭颖

收件人: linqisheng074

Re: Confirm our revised author list

今天 傍晚6:26

Confirmed
Xuying Zhu

---- Replied Message ----

| | |
|---|---|
| From | Lin Qisheng<linqisheng074@hotmail.com> |
| Date | 01/06/2024 17:54 |
| To | xuyingzh1@163.com |
| Subject | Confirm our revised author list |

Dear All,

Our manuscript ' DDRGK1-mediated ER-phagy attenuates acute kidney injury through ER-stress and apoptosis ' will be accepted on *Cell Death Dis* soon. Dr Shan Wu helps us during the major revision period, and is listed as co-author on our manscript.

Please email me if you confirm the chan[illegible] our manuscript.

回复 全部回复 转发 删除 更多

Yin Zhou

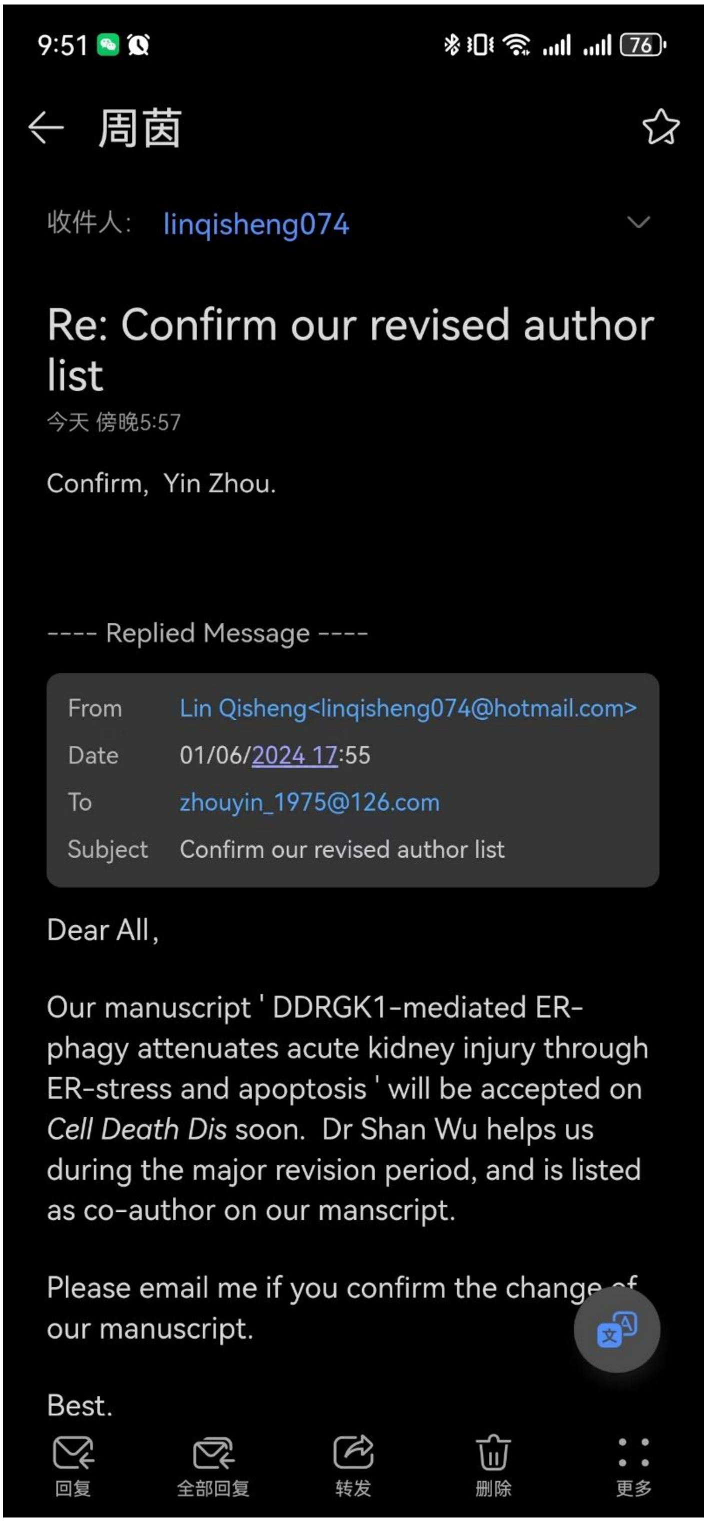

9:51

周茵

收件人: linqisheng074

Re: Confirm our revised author list

今天 傍晚5:57

Confirm, Yin Zhou.

---- Replied Message ----

| | |
|---|---|
| From | Lin Qisheng<linqisheng074@hotmail.com> |
| Date | 01/06/2024 17:55 |
| To | zhouyin_1975@126.com |
| Subject | Confirm our revised author list |

Dear All,

Our manuscript ' DDRGK1-mediated ER-phagy attenuates acute kidney injury through ER-stress and apoptosis ' will be accepted on *Cell Death Dis* soon. Dr Shan Wu helps us during the major revision period, and is listed as co-author on our manscript.

Please email me if you confirm the change of our manuscript.

Best.

回复 全部回复 转发 删除 更多

Yao Xu

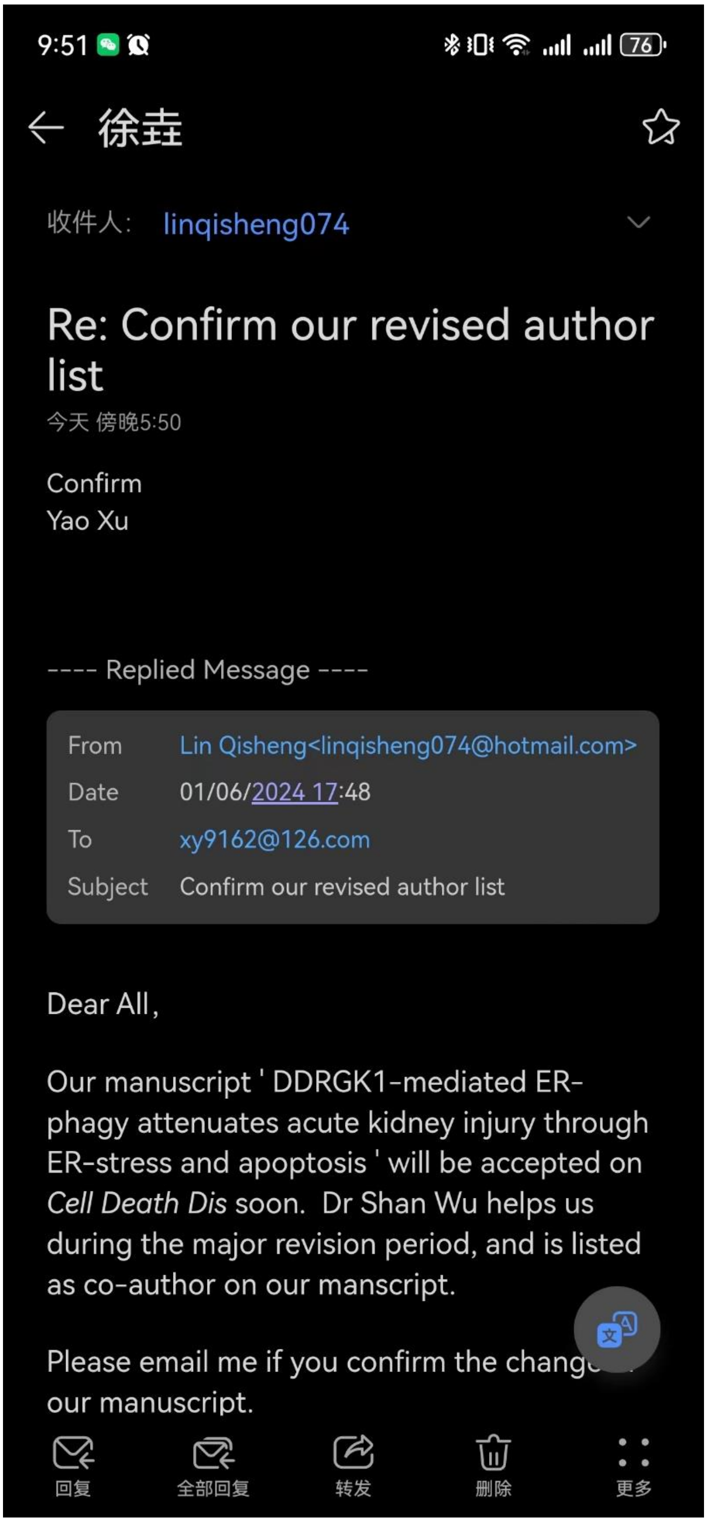

9:51

← 徐垚

收件人: linqisheng074

Re: Confirm our revised author list

今天 傍晚5:50

Confirm
Yao Xu

---- Replied Message ----

| | |
| --- | --- |
| From | Lin Qisheng<linqisheng074@hotmail.com> |
| Date | 01/06/2024 17:48 |
| To | xy9162@126.com |
| Subject | Confirm our revised author list |

Dear All,

Our manuscript ' DDRGK1-mediated ER-phagy attenuates acute kidney injury through ER-stress and apoptosis ' will be accepted on *Cell Death Dis* soon.  Dr Shan Wu helps us during the major revision period, and is listed as co-author on our manscript.

Please email me if you confirm the chang[illegible] our manuscript.

回复 全部回复 转发 删除 更多

Jialin Li

9:51

李佳琳

收件人: Lin Qisheng

# Re: Confirm our revised author list

今天 傍晚6:12

Dear Lin,

I have confirmed the change of our manuscript.

Best,
Jialin Li

---- Replied Message ----

| | |
|---|---|
| From | Lin Qisheng<linqisheng074@hotmail.com> |
| Date | 01/06/2024 18:04 |
| To | miffy429@126.com<miffy429@126.com> |
| Subject | Confirm our revised author list |

Dear All,

Our manuscript ' DDRGK1-mediated ER-phagy attenuates acute kidney injury through ER-stress and apoptosis ' will be accepted on *Cell Death Dis* soon. Dr Shan Wu helps u[illegible] during the major revision period, and is [illegible] as co-author on our manscript.

回复 全部回复 转发 删除 更多

Chaojun Qi

9:50

Qichaojun

收件人: Lin Qisheng

## Re: Confirm our revised author list

今天 傍晚6:27

Agree.

Chaojun Qi

在 2024年1月6日，18:06，Lin Qisheng
<linqisheng074@hotmail.com> 写道:

Dear All,

Our manuscript ' DDRGK1-mediated ER-phagy attenuates acute kidney injury through ER-stress and apoptosis ' will be accepted on *Cell Death Dis* soon. Dr Shan Wu helps us during the major revision period, and is listed as co-author on our manscript.

Please email me if you confirm the change of our manuscript.

Best,

Qisheng Lin

--

回复 全部回复 转发 删除 更多

Xinghua Shao

9:50 76

邵兴华

收件人: Lin Qisheng

## Re:Confirm our revised author list

今天 傍晚6:29

ok, Xinghua Shao

At 2024-01-06 18:07:28, "Lin Qisheng" <linqisheng074@hotmail.com> wrote:

Dear All,

Our manuscript ' DDRGK1-mediated ER-phagy attenuates acute kidney injury through ER-stress and apoptosis ' will be accepted on *Cell Death Dis* soon. Dr Shan Wu helps us during the major revision period, and is listed as co-author on our manscript.

Please email me if you confirm the change of our manuscript.

Best,

Qisheng Lin

回复 全部回复 转发 删除 更多

Jingkui Wu

9:50

wjk_health

收件人: linqisheng074

# Re: Confirm our revised author list

今天 傍晚6:24

ok  Jingkui Wu

---- Replied Message ----

| | |
|---|---|
| From | Lin Qisheng<linqisheng074@hotmail.com> |
| Date | 01/06/2024 18:08 |
| To | wjk_health@163.com |
| Subject | Confirm our revised author list |

Dear All,

Our manuscript ' DDRGK1-mediated ER-phagy attenuates acute kidney injury through ER-stress and apoptosis ' will be accepted on *Cell Death Dis* soon.  Dr Shan Wu helps us during the major revision period, and is listed as co-author on our manscript.

Please email me if you confirm the change of our manuscript.

Best.

回复 全部回复 转发 删除 更多

Shan Wu

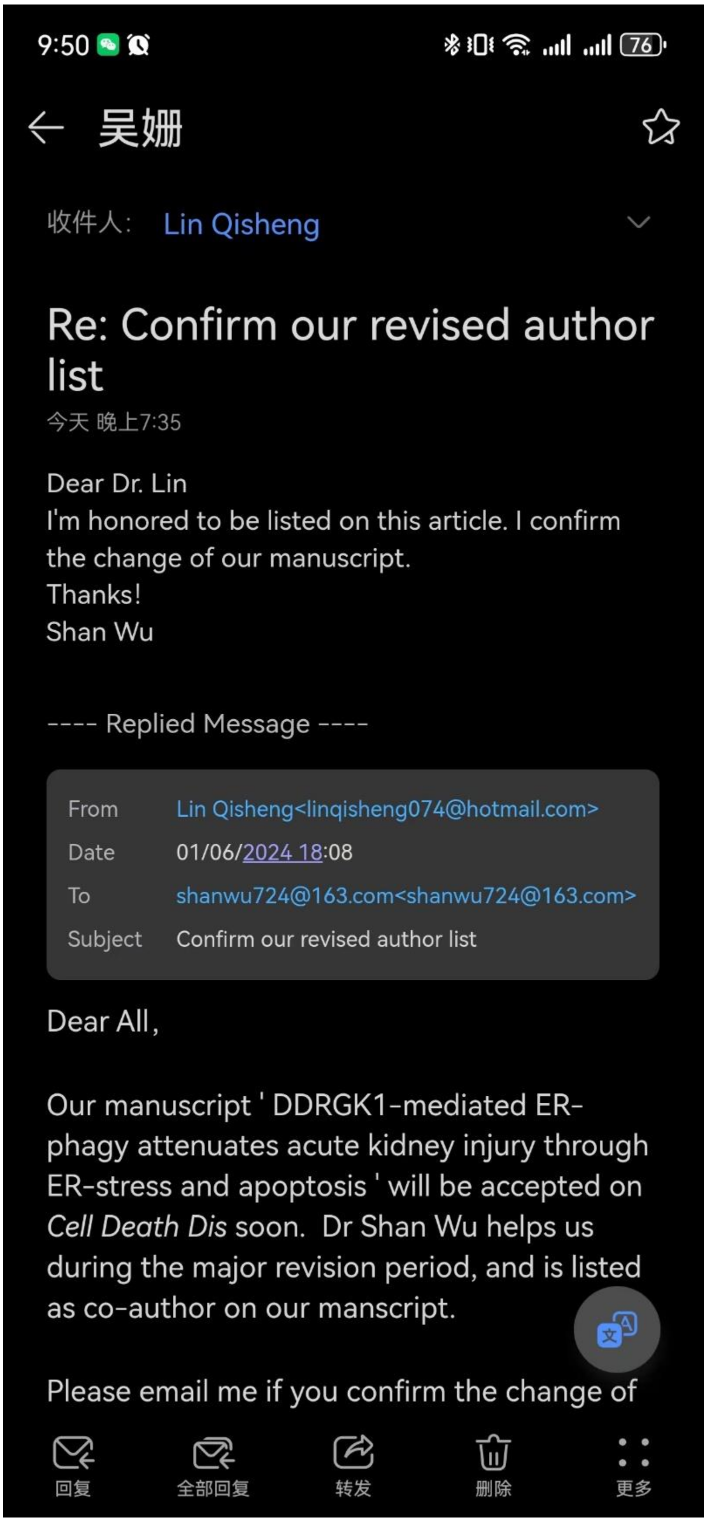

9:50

吴姗

收件人: Lin Qisheng

Re: Confirm our revised author list

今天 晚上7:35

Dear Dr. Lin
I'm honored to be listed on this article. I confirm the change of our manuscript.
Thanks!
Shan Wu

---- Replied Message ----

| | |
|---|---|
| From | Lin Qisheng<linqisheng074@hotmail.com> |
| Date | 01/06/2024 18:08 |
| To | shanwu724@163.com<shanwu724@163.com> |
| Subject | Confirm our revised author list |

Dear All,

Our manuscript ' DDRGK1-mediated ER-phagy attenuates acute kidney injury through ER-stress and apoptosis ' will be accepted on *Cell Death Dis* soon. Dr Shan Wu helps us during the major revision period, and is listed as co-author on our manscript.

Please email me if you confirm the change of

回复 全部回复 转发 删除 更多

Hong Cai

8:58

Hong Cai

收件人: Lin Qisheng

回复:Confirm our revised author list

今天 晚上8:44

Yes，I confirm the change of our manuscript.

----- 原始邮件 -----

**发件人：** Lin Qisheng<linqisheng074@hotmail.com>

**发送时间：** 2024-01-06 18:10:22

**收件人：** chcaicai@sohu.com<chcaicai@sohu.com>

**主 题：** Confirm our revised author list

Dear All,

Our manuscript ' DDRGK1-mediated ER-phagy attenuates acute kidney injury through ER-stress and apoptosis ' will be accepted on *Cell Death Dis* soon. Dr Shan Wu helps us during the major revision period, and is listed as co-author on our manscript.

Please email me if you confirm the chang[illegible] our manuscript.

回复 全部回复 转发 删除 更多

Leyi Gu

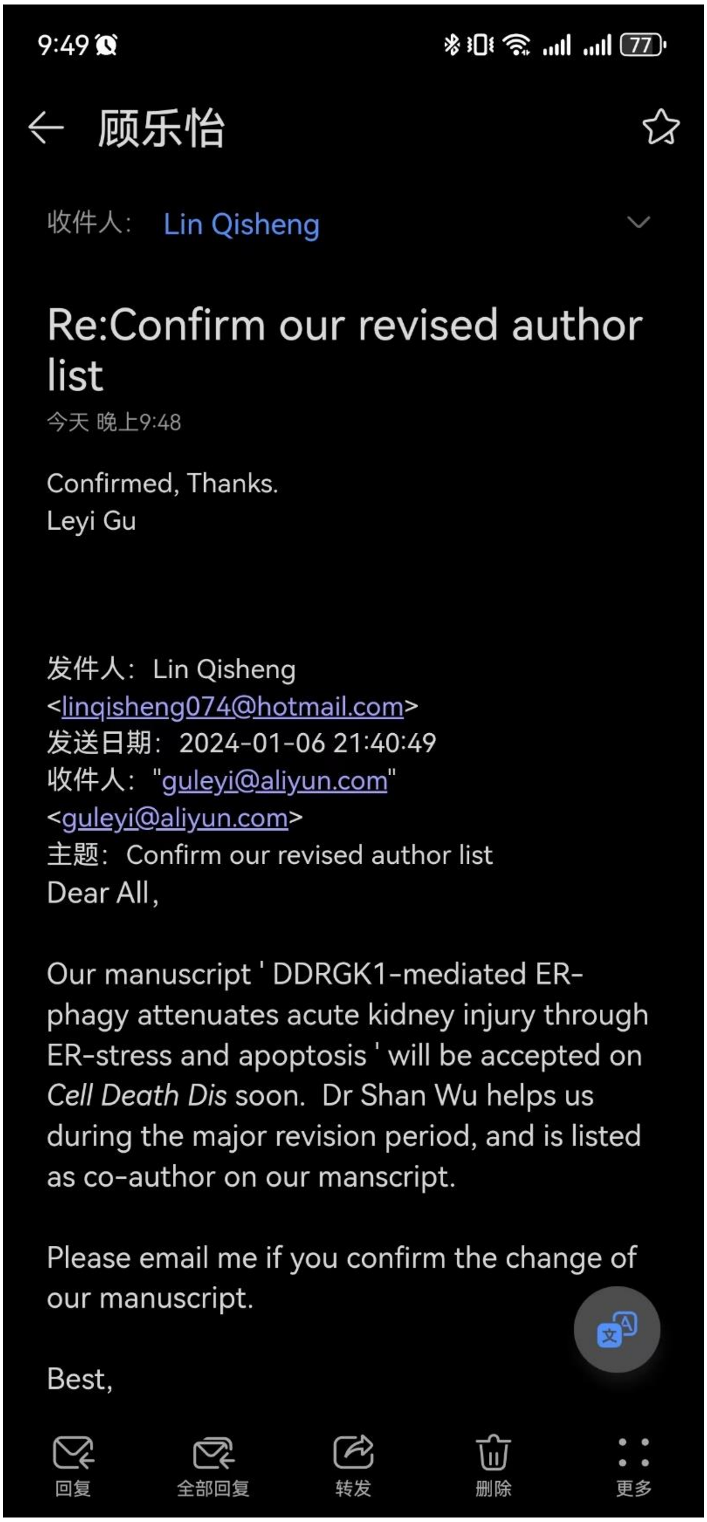

9:49

顾乐怡

收件人： Lin Qisheng

Re:Confirm our revised author list

今天 晚上9:48

Confirmed, Thanks.
Leyi Gu

发件人：Lin Qisheng
<linqisheng074@hotmail.com>
发送日期：2024-01-06 21:40:49
收件人："guleyi@aliyun.com"
<guleyi@aliyun.com>
主题：Confirm our revised author list
Dear All,

Our manuscript ' DDRGK1-mediated ER-phagy attenuates acute kidney injury through ER-stress and apoptosis ' will be accepted on *Cell Death Dis* soon. Dr Shan Wu helps us during the major revision period, and is listed as co-author on our manscript.

Please email me if you confirm the change of our manuscript.

Best,

回复 全部回复 转发 删除 更多

Shan Mou

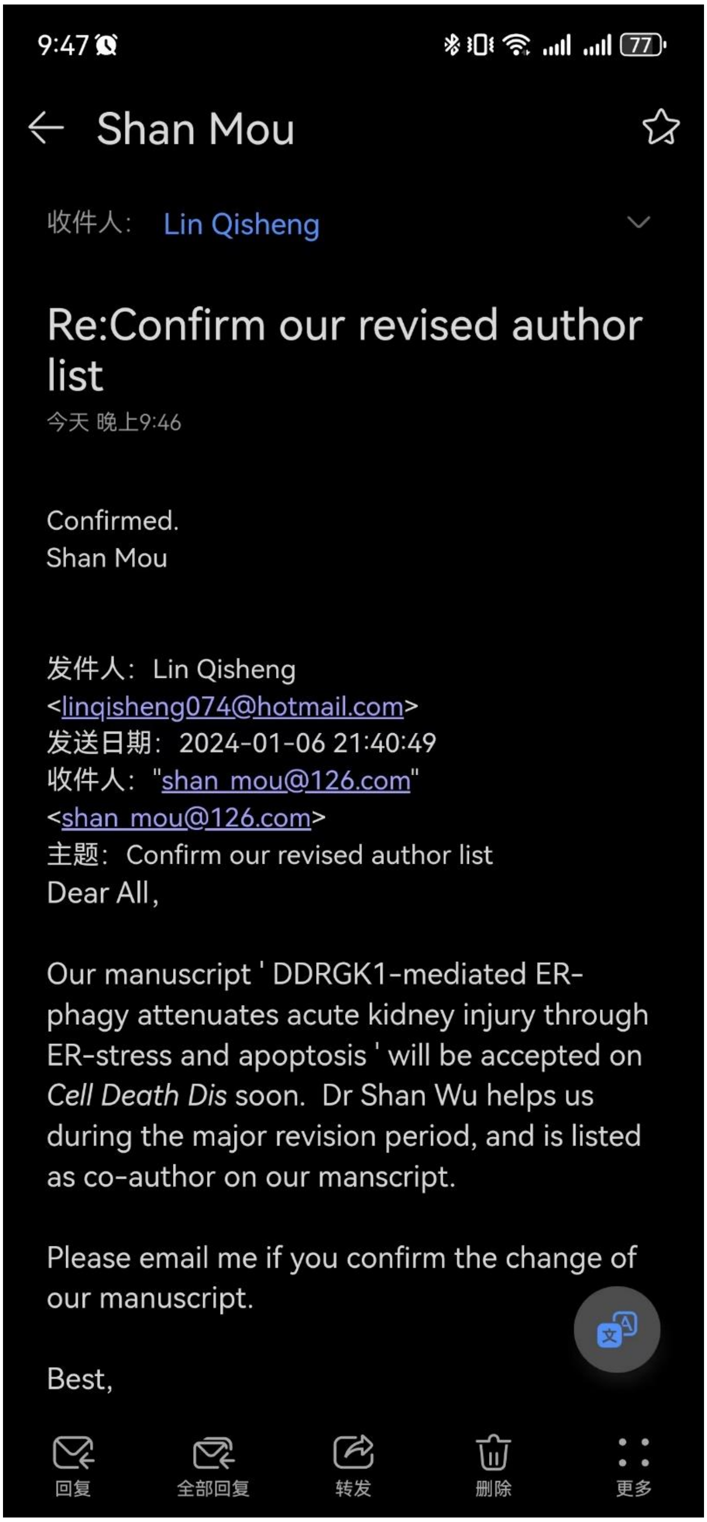

9:47

Shan Mou

收件人： Lin Qisheng

Re:Confirm our revised author list

今天 晚上9:46

Confirmed.
Shan Mou

发件人： Lin Qisheng
<linqisheng074@hotmail.com>
发送日期： 2024-01-06 21:40:49
收件人： "shan_mou@126.com"
<shan_mou@126.com>
主题： Confirm our revised author list

Dear All,

Our manuscript ' DDRGK1-mediated ER-phagy attenuates acute kidney injury through ER-stress and apoptosis ' will be accepted on *Cell Death Dis* soon. Dr Shan Wu helps us during the major revision period, and is listed as co-author on our manscript.

Please email me if you confirm the change of our manuscript.

Best,

回复 全部回复 转发 删除 更多

Zhaohui Ni

9:40

# Zhaohui Ni

收件人: Lin Qisheng

## Re:Confirm our revised author list

今天 晚上9:39

OK
Zhaohui Ni

At 2024-01-06 18:36:19, "Lin Qisheng" <linqisheng074@hotmail.com> wrote:

Dear All ,

Our manuscript ' DDRGK1-mediated ER-phagy attenuates acute kidney injury through ER-stress and apoptosis ' will be accepted on *Cell Death Dis* soon.  Dr Shan Wu helps us during the major revision period, and is listed as co-author on our manscript.

Please email me if you confirm the change of our manuscript.

Best,

回复 全部回复 转发 删除 更多

Shu Li

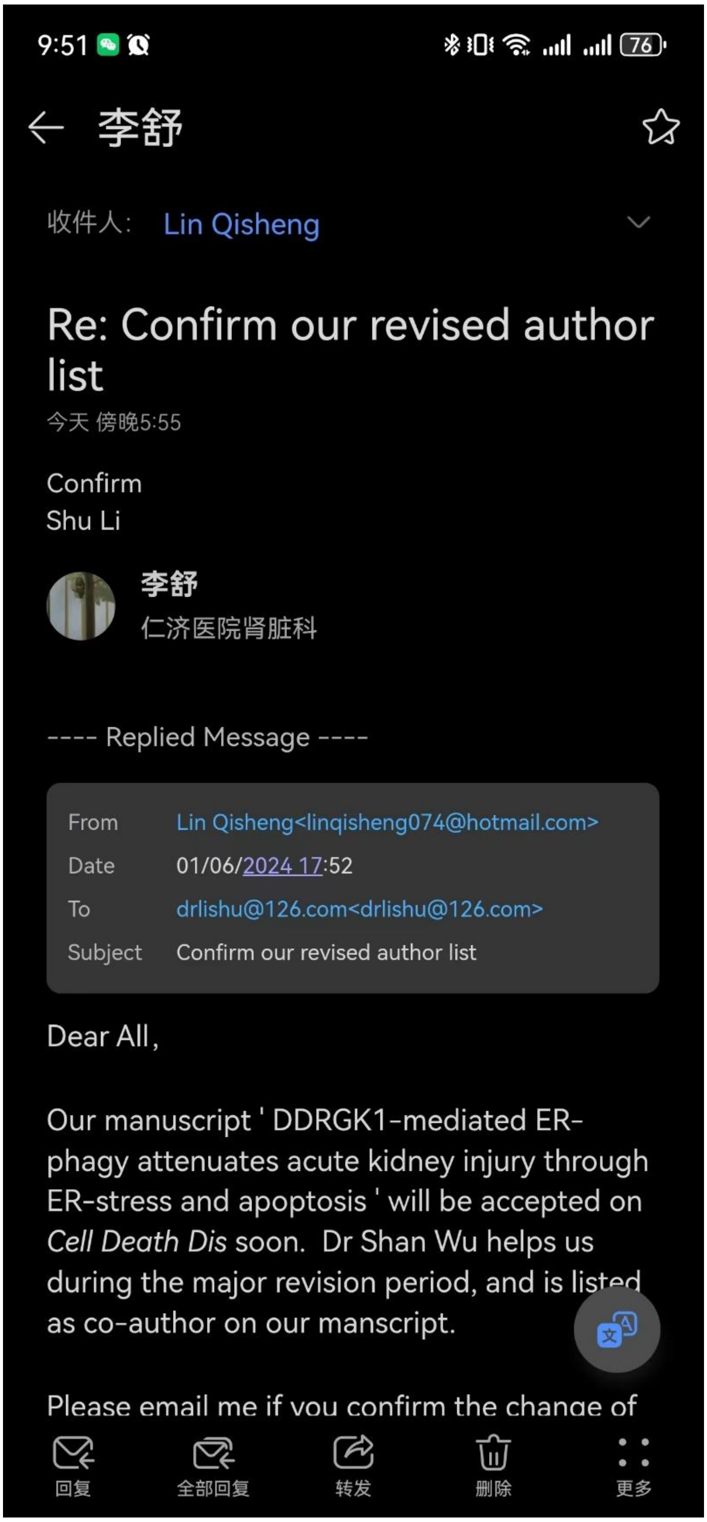

9:51

← 李舒

收件人: Lin Qisheng

Re: Confirm our revised author list

今天 傍晚5:55

Confirm
Shu Li

李舒
仁济医院肾脏科

---- Replied Message ----

| | |
|---|---|
| From | Lin Qisheng<linqisheng074@hotmail.com> |
| Date | 01/06/2024 17:52 |
| To | drlishu@126.com<drlishu@126.com> |
| Subject | Confirm our revised author list |

Dear All,

Our manuscript ' DDRGK1-mediated ER-phagy attenuates acute kidney injury through ER-stress and apoptosis ' will be accepted on *Cell Death Dis* soon. Dr Shan Wu helps us during the major revision period, and is listed as co-author on our manscript.

Please email me if you confirm the change of

回复 全部回复 转发 删除 更多

Qisheng Lin

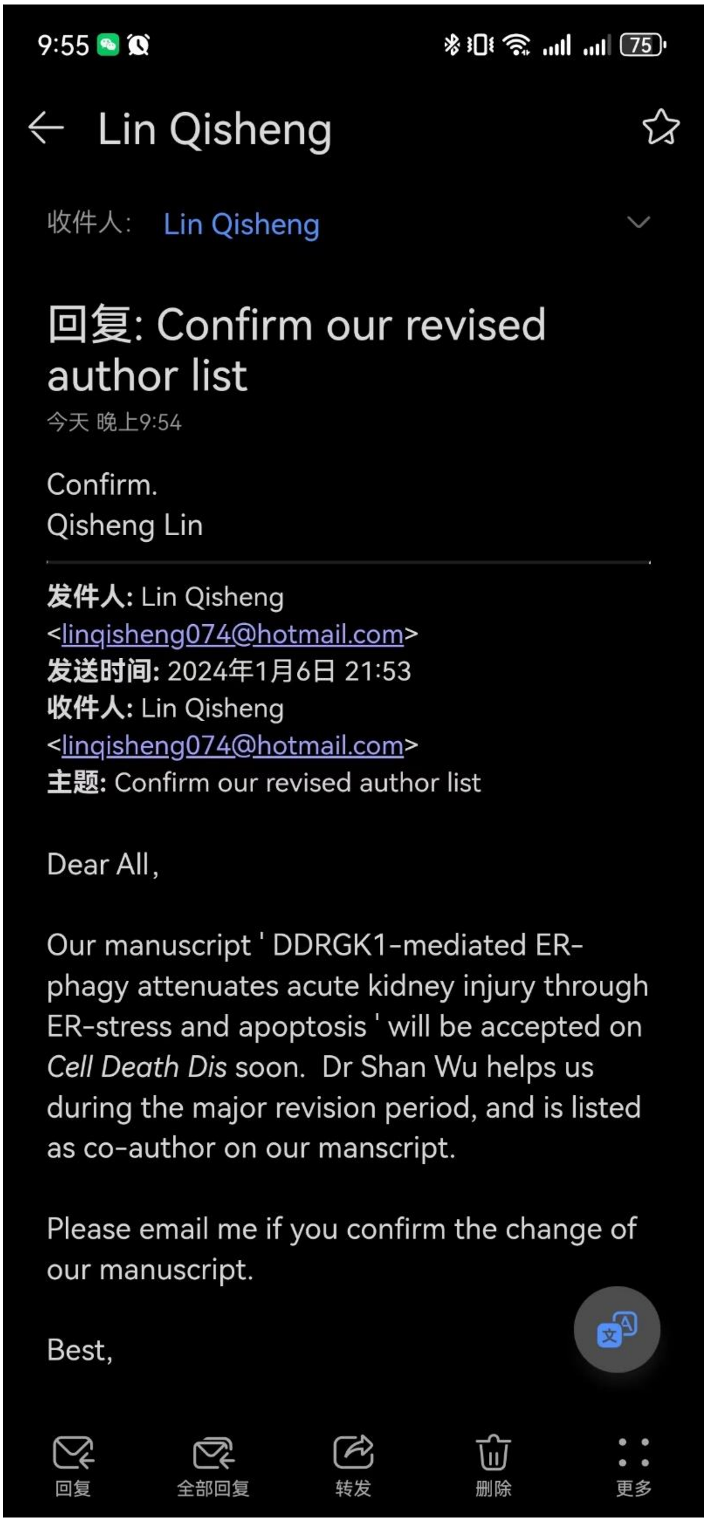

9:55

Lin Qisheng

收件人: Lin Qisheng

回复: Confirm our revised author list

今天 晚上9:54

Confirm.
Qisheng Lin

**发件人:** Lin Qisheng <linqisheng074@hotmail.com>
**发送时间:** 2024年1月6日 21:53
**收件人:** Lin Qisheng <linqisheng074@hotmail.com>
**主题:** Confirm our revised author list

Dear All,

Our manuscript ' DDRGK1-mediated ER-phagy attenuates acute kidney injury through ER-stress and apoptosis ' will be accepted on *Cell Death Dis* soon. Dr Shan Wu helps us during the major revision period, and is listed as co-author on our manscript.

Please email me if you confirm the change of our manuscript.

Best,

回复 全部回复 转发 删除 更多
