# Supplementary material for: DDRGK1-mediated ER-phagy attenuates acute kidney injury through ER-stress and apoptosis
Source: Cell Death Dis. 2024 Jan 17;15(1):63. doi: 10.1038/s41419-024-06449-4 (PMC10794694; doi:10.1038/s41419-024-06449-4)

Fig2

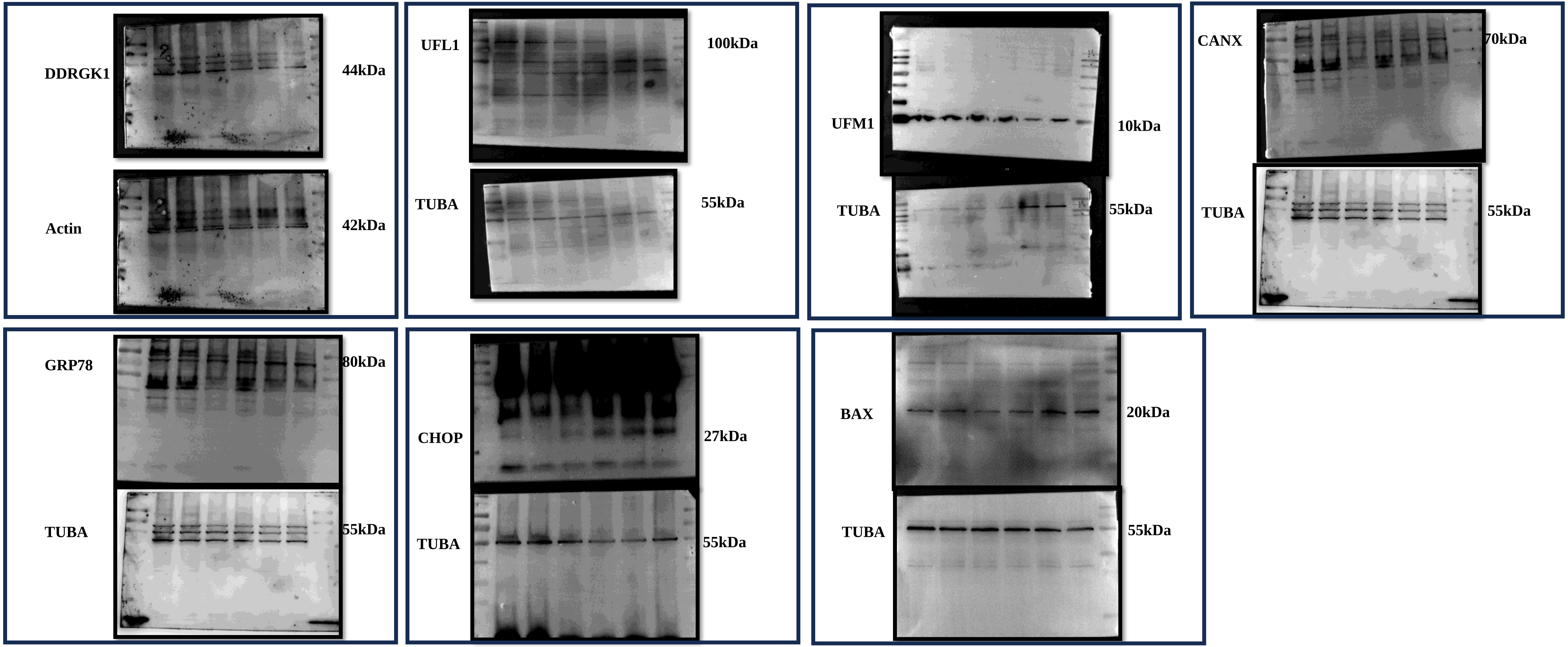
DDRGK1
44kDa
Actin
42kDa
UFL1
100kDa
TUBA
55kDa
UFM1
10kDa
TUBA
55kDa
CANX
70kDa
TUBA
55kDa
GRP78
80kDa
TUBA
55kDa
CHOP
27kDa
TUBA
55kDa
BAX
20kDa
TUBA
55kDa

Fig3

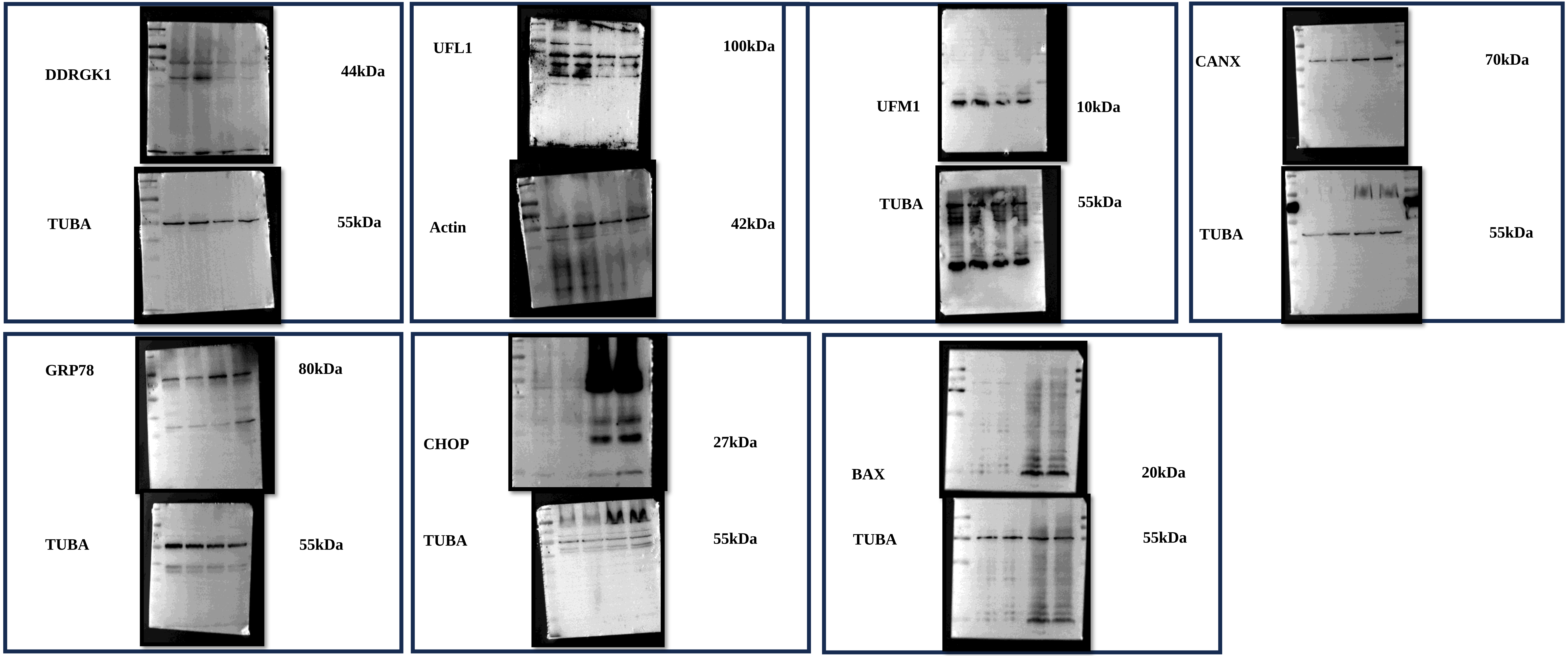
DDRGK1
44kDa
TUBA
55kDa
UFL1
100kDa
Actin
42kDa
UFM1
10kDa
TUBA
55kDa
CANX
70kDa
TUBA
55kDa
GRP78
80kDa
TUBA
55kDa
CHOP
27kDa
TUBA
55kDa
BAX
20kDa
TUBA
55kDa

Fig4

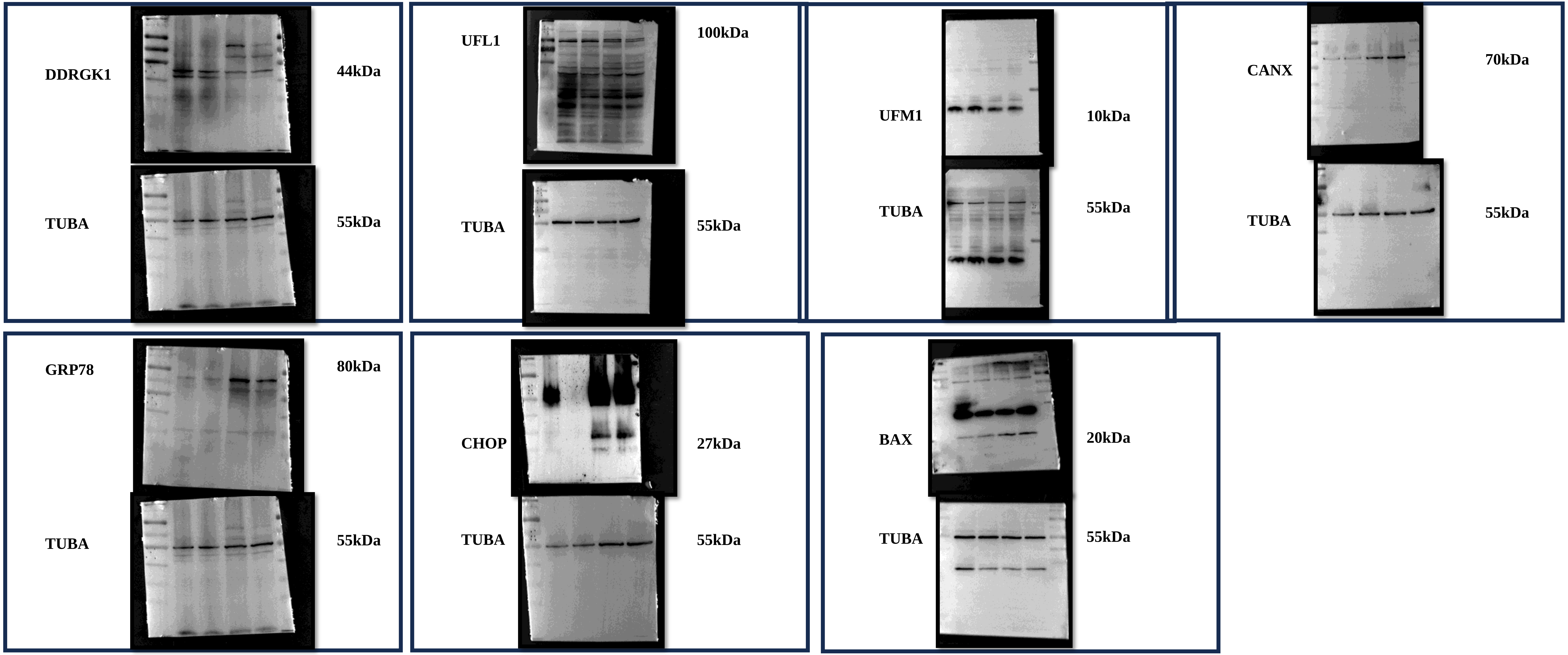

DDRGK1
44kDa
TUBA
55kDa
UFL1
100kDa
TUBA
55kDa
UFM1
10kDa
TUBA
55kDa
CANX
70kDa
TUBA
55kDa
GRP78
80kDa
TUBA
55kDa
CHOP
27kDa
TUBA
55kDa
BAX
20kDa
TUBA
55kDa

Fig5

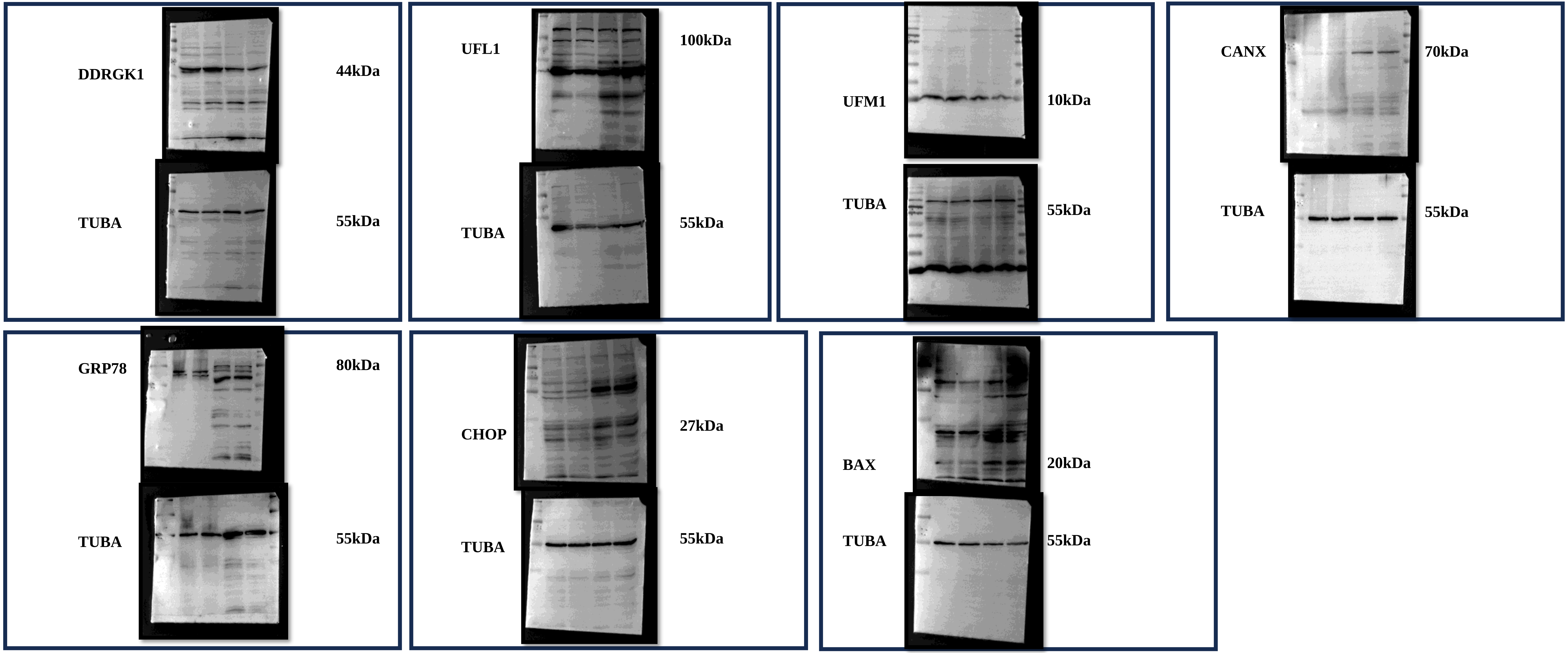
DDRGK1
44kDa
TUBA
55kDa
UFL1
100kDa
TUBA
55kDa
UFM1
10kDa
TUBA
55kDa
CANX
70kDa
TUBA
55kDa
GRP78
80kDa
TUBA
55kDa
CHOP
27kDa
TUBA
55kDa
BAX
20kDa
TUBA
55kDa

Fig6

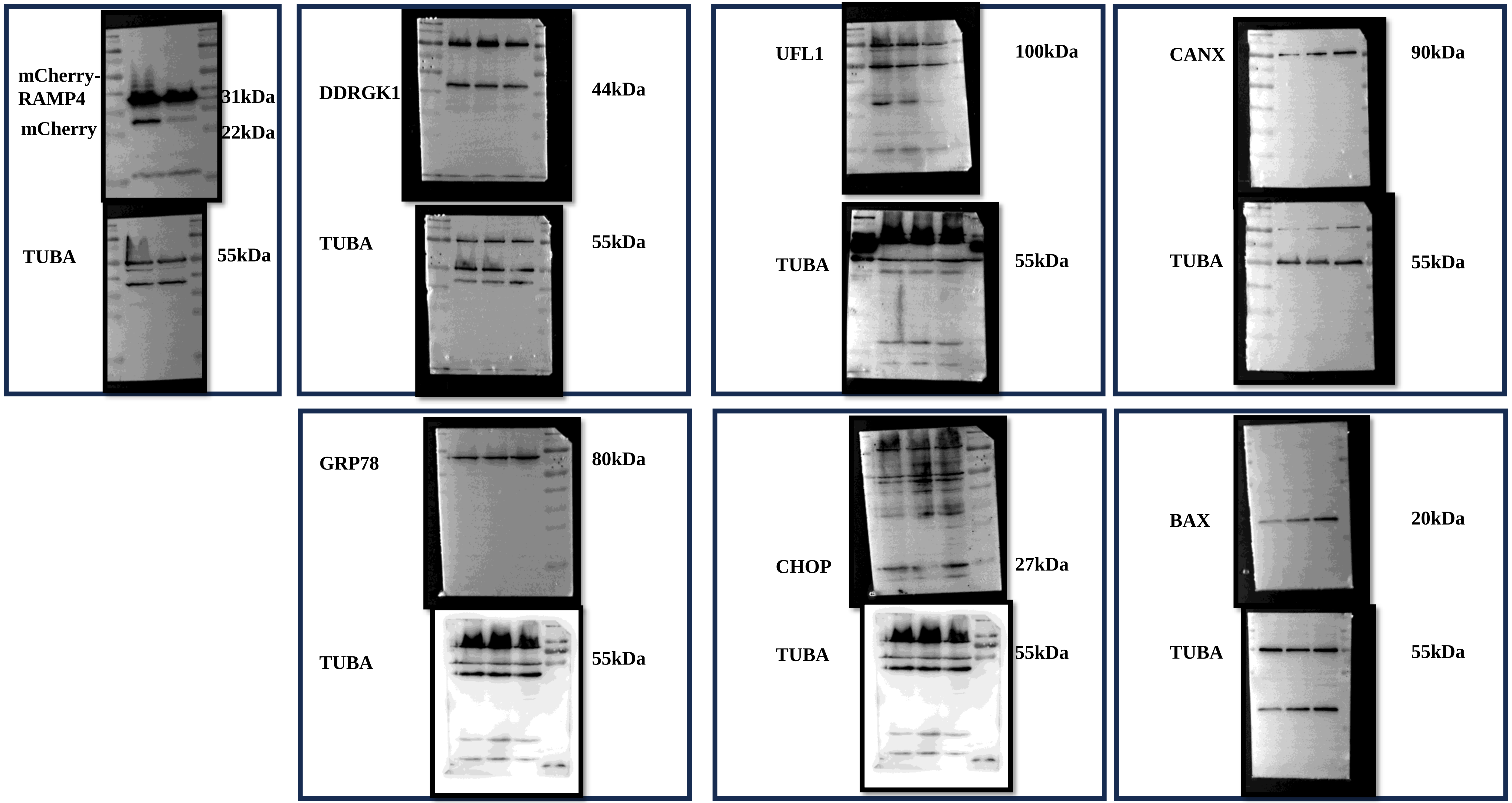
mCherry-RAMP4
mCherry
31kDa
22kDa
TUBA
55kDa
DDRGK1
44kDa
TUBA
55kDa
UFL1
100kDa
TUBA
55kDa
CANX
90kDa
TUBA
55kDa
GRP78
80kDa
TUBA
55kDa
CHOP
27kDa
TUBA
55kDa
BAX
20kDa
TUBA
55kDa

Fig7

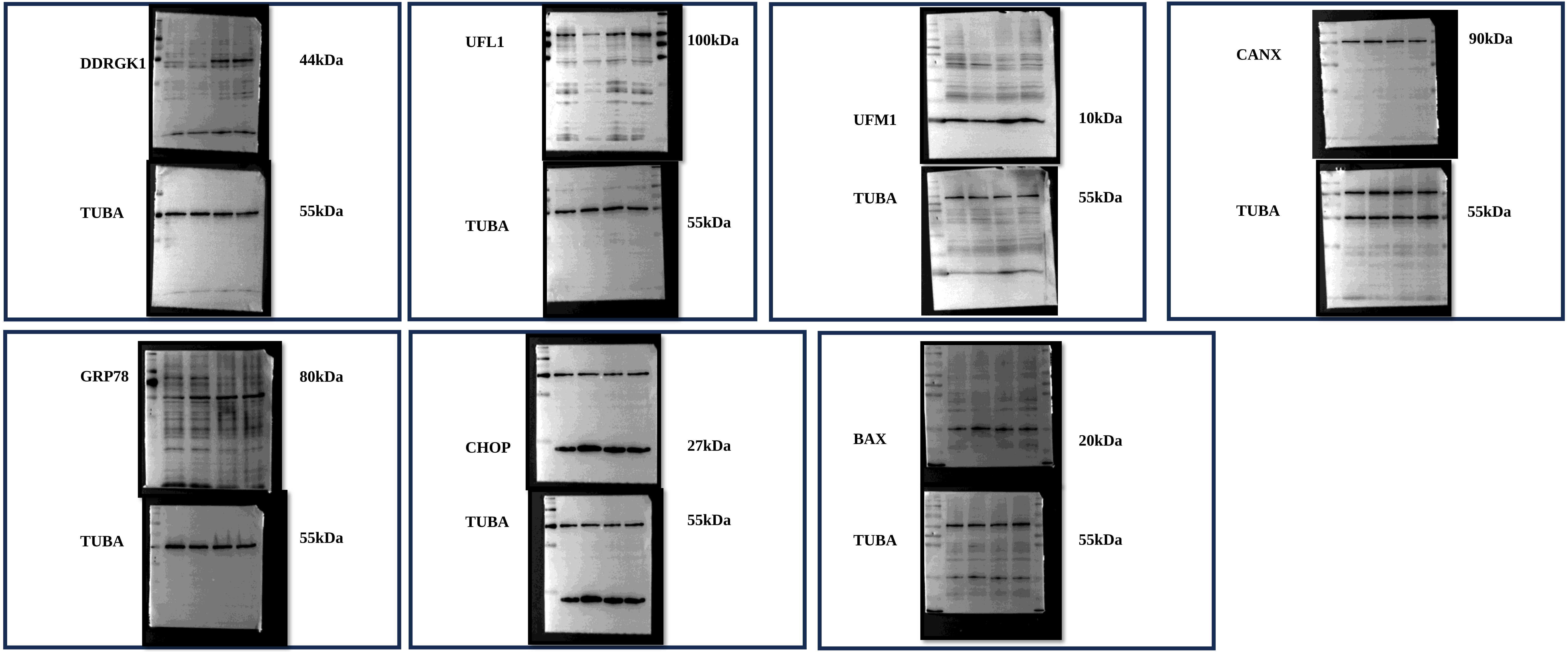
DDRGK1
44kDa
TUBA
55kDa
UFL1
100kDa
TUBA
55kDa
UFM1
10kDa
TUBA
55kDa
CANX
90kDa
TUBA
55kDa
GRP78
80kDa
TUBA
55kDa
CHOP
27kDa
TUBA
55kDa
BAX
20kDa
TUBA
55kDa

Fig8

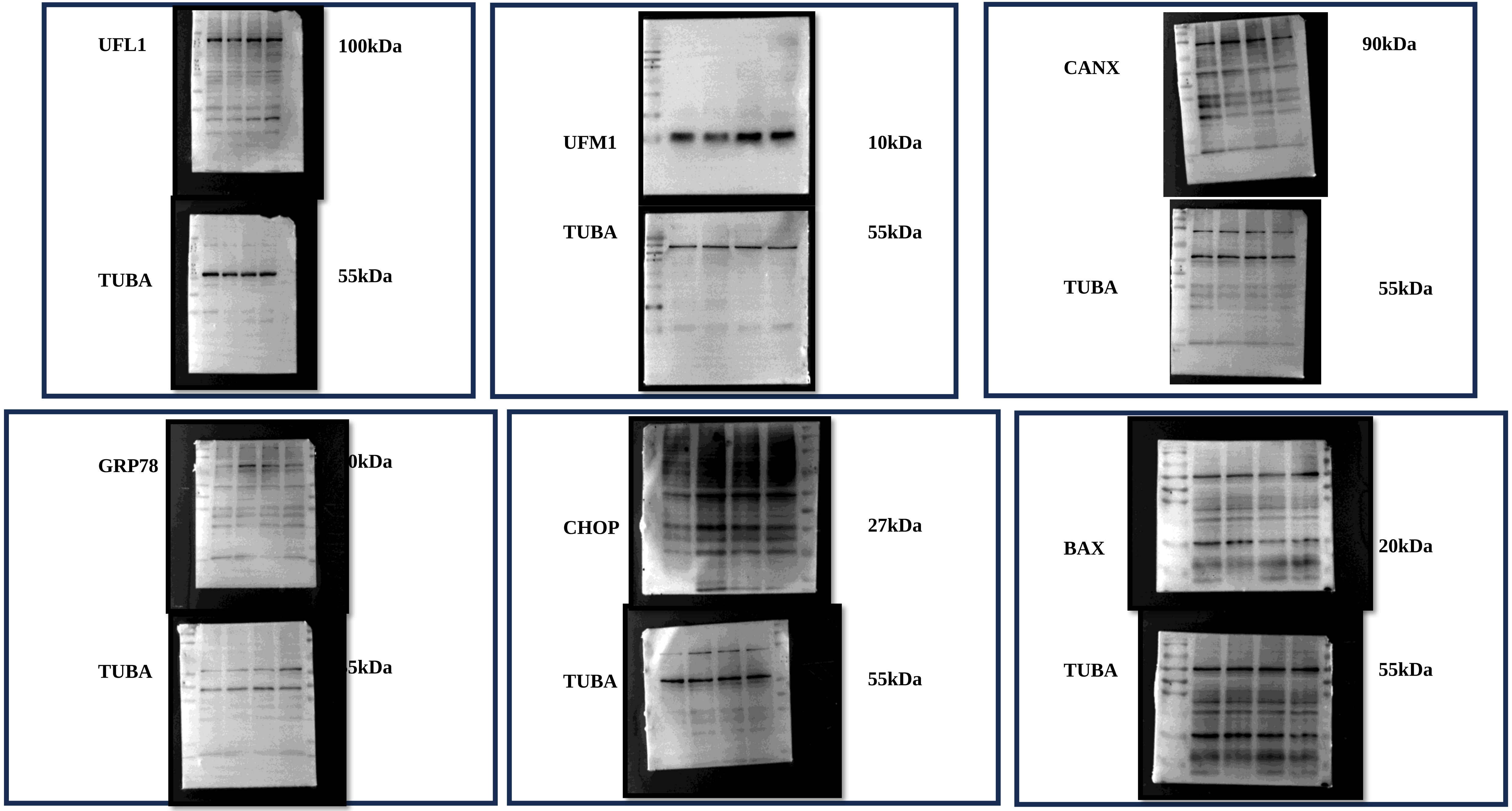
UFL1
100kDa
TUBA
55kDa
UFM1
10kDa
TUBA
55kDa
CANX
90kDa
TUBA
55kDa
GRP78
0kDa
TUBA
5kDa
CHOP
27kDa
TUBA
55kDa
BAX
20kDa
TUBA
55kDa

Supplemental Fig

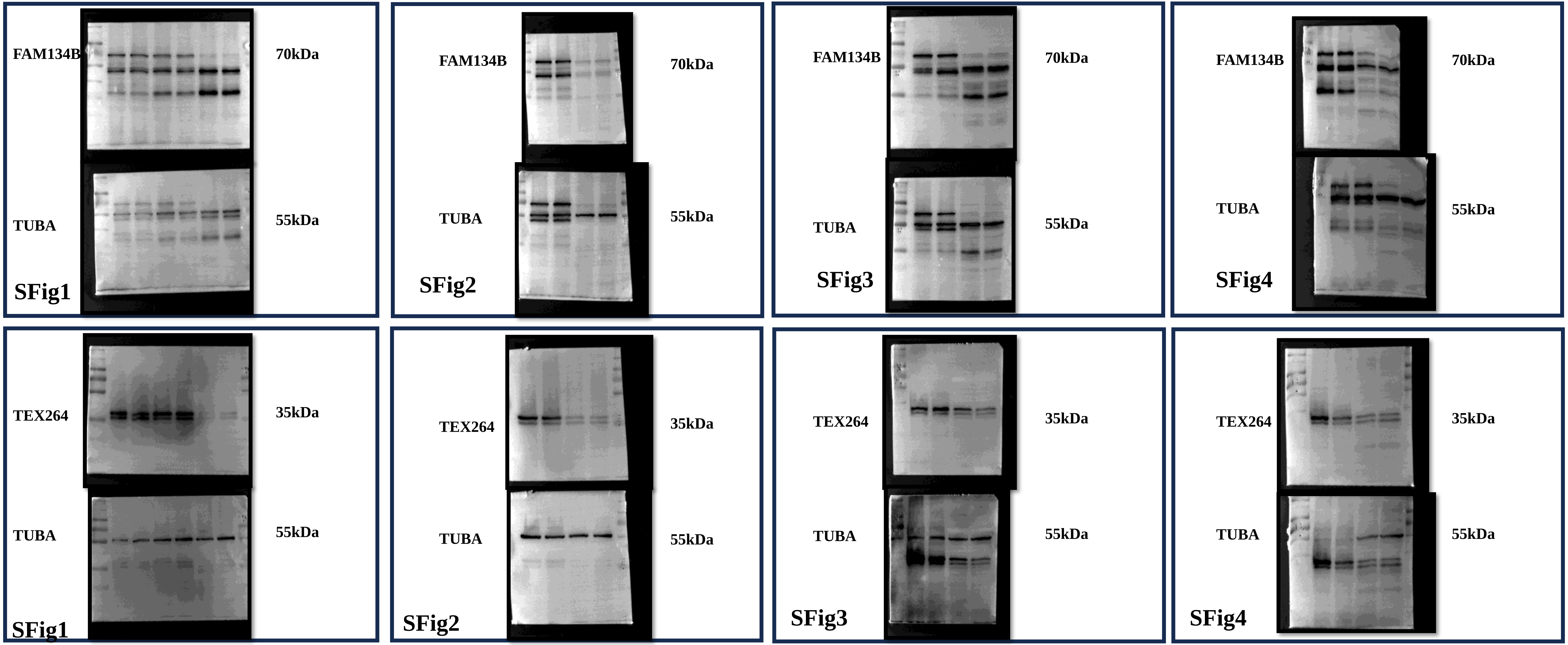

Supplemental Fig

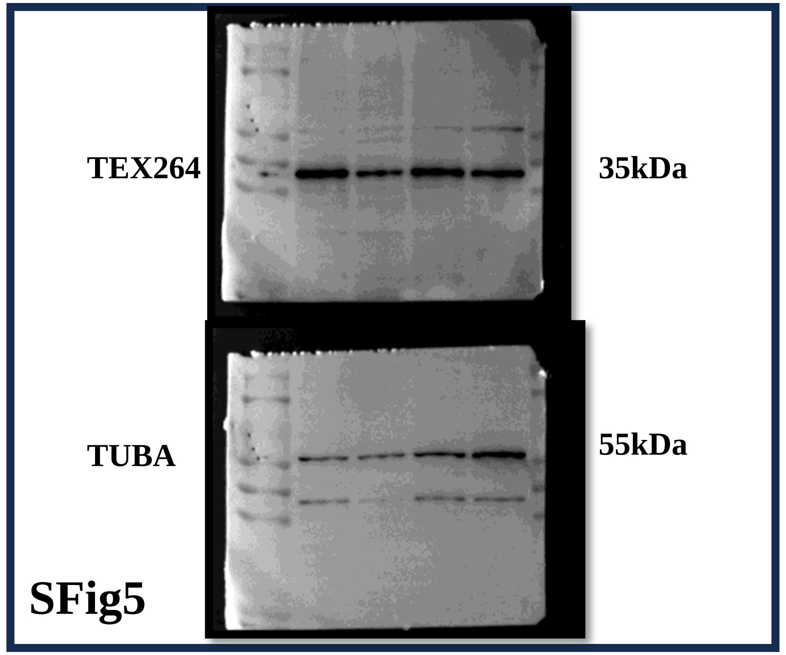

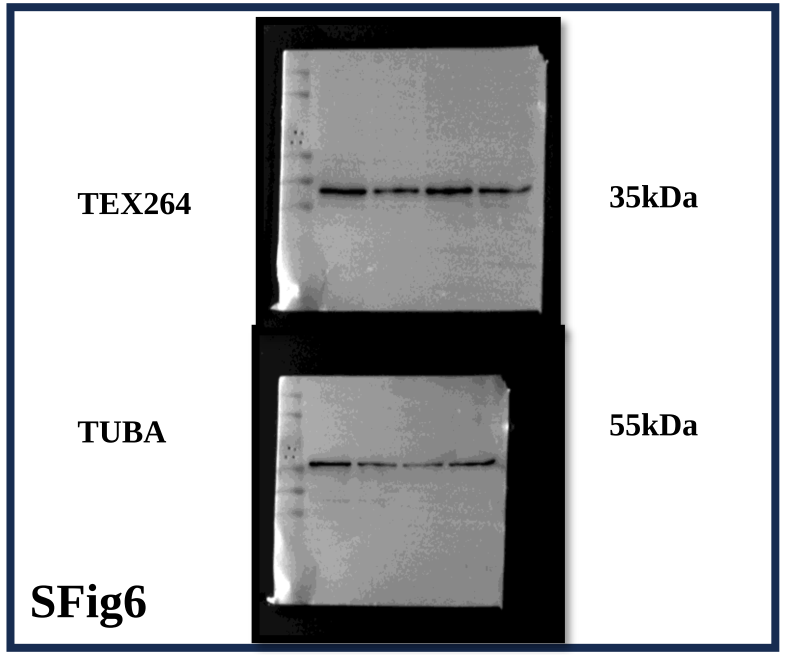

Supplement: Supplementary file 2 — Supplementary File-Original western blots [file 41419_2024_6449_MOESM2_ESM.pdf]
