# Supplementary material for: DDRGK1-mediated ER-phagy attenuates acute kidney injury through ER-stress and apoptosis
Source: Cell Death Dis. 2024 Jan 17;15(1):63. doi: 10.1038/s41419-024-06449-4 (PMC10794694; doi:10.1038/s41419-024-06449-4)
Supplement: Supplementary file 3 — Supplemental Figure 1-6 [file 41419_2024_6449_MOESM3_ESM.pdf]

Supplemental Figure

Supplemental Fig1

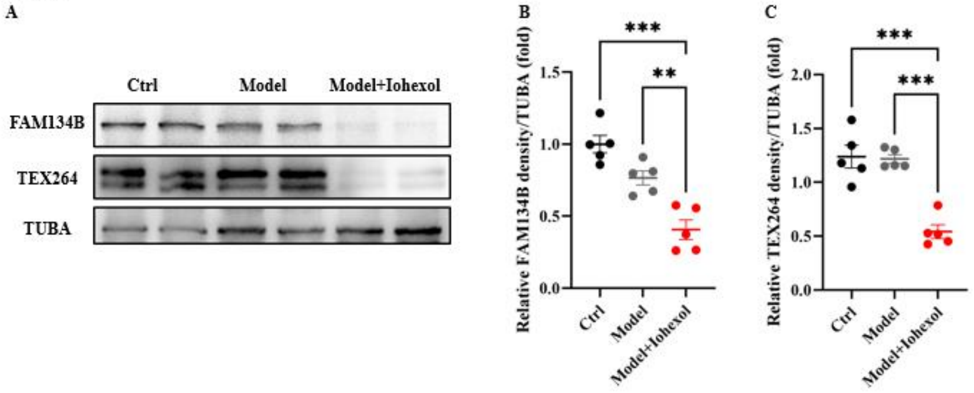

Supplemental Figure1. FAM134B and TEX264 in CI-AKI mice. (A-C) The immunoblot analysis and quantification of FAM134B and TEX264 in kidney lysates. Data are presented as the mean ± SEM (n=5). **p<0.01 and ***p<0.001.

Supplemental Fig2

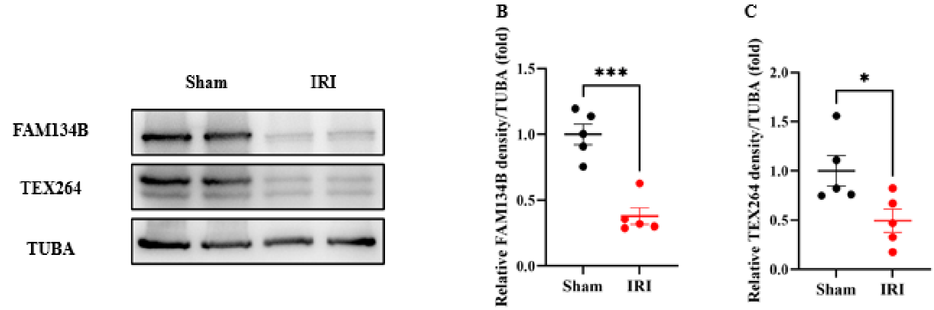

Supplemental Figure2. FAM134B and TEX264 in IRI mice. (A-C) The immunoblot analysis and quantification of FAM134B and TEX264 in kidney lysates. Data are presented as the mean ± SEM (n=5). *p<0.05 and ***p<0.001.

Supplemental Fig3

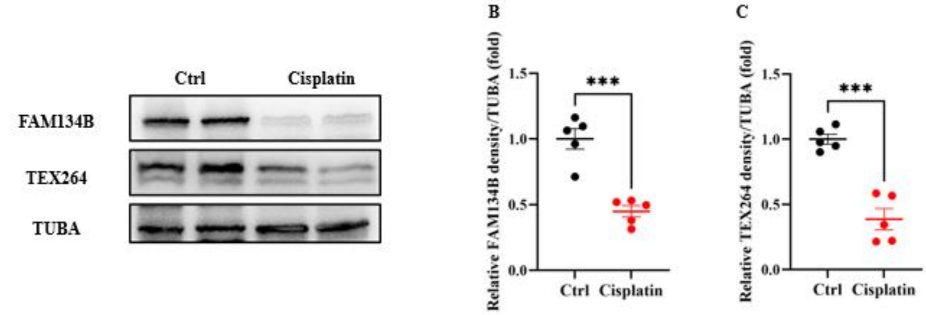

Supplemental Figure3. FAM134B and TEX264 in Cisplatin mice. (A-C) The immunoblot analysis and quantification of FAM134B and TEX264 in kidney lysates. Data are presented as the mean ± SEM (n=5). ***p<0.001.

Supplemental Fig4

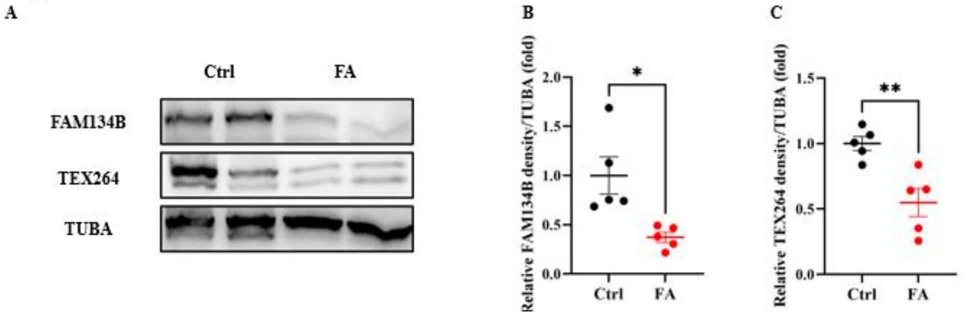

Supplemental Figure4. FAM134B and TEX264 in FA mice. (A-C) The immunoblot analysis and quantification of FAM134B and TEX264 in kidney lysates. Data are presented as the mean ± SEM (n=5). *p<0.05 and **p<0.01.

Supplemental Fig5

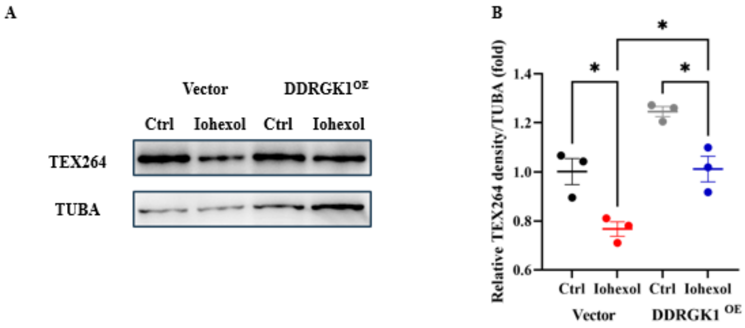

Supplemental Figure5. TEX264 in *DDRGK1* overexpression HK-2 cells. (A, B) The immunoblot analysis and quantification of TEX264 in cell lysates. Data are presented as the mean ± SEM (n=3). *p<0.05.

Supplemental Fig6

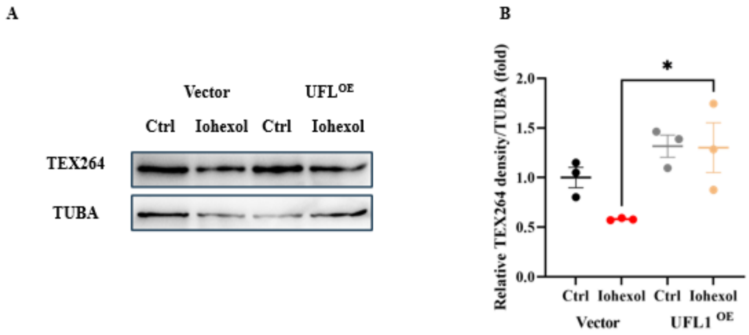

Supplemental Figure6. TEX264 in *UFL1* overexpression HK-2 cells. (A, B) The immunoblot analysis and quantification of TEX264 in cell lysates. Data are presented as the mean ± SEM (n=3). *p<0.05.
